# Isolation and Structural Elucidation of Chondrosterins F–H from the Marine Fungus *Chondrostereum* sp

**DOI:** 10.3390/md11020551

**Published:** 2013-02-22

**Authors:** Hou-Jin Li, Ting Chen, Ying-Lu Xie, Wen-Dan Chen, Xiao-Feng Zhu, Wen-Jian Lan

**Affiliations:** 1 School of Chemistry and Chemical Engineering, Sun Yat-Sen University, Guangzhou 510275, China; E-Mails: ceslhj@mail.sysu.edu.cn (H.-J.L.); zsulhj@yahoo.com (T.C.); natprod3698@gmail.com (Y.-L.X.); 2 State Key Laboratory of Oncology in South China, Cancer Center, Sun Yat-Sen University, Guangzhou 510060, China; E-Mails: chenwd@sysucc.org.cn (W.-D.C.); zhuxfeng@mail.sysu.edu.cn (X.-F.Z.); 3 School of Pharmaceutical Sciences, Sun Yat-Sen University, Guangzhou 510006, China; 4 Guangdong Technology Research Center for Advanced Chinese Medicine, Guangzhou 510006, China

**Keywords:** *Chondrostereum* sp., sesquiterpenoids, chondrosterins, marine fungus, cytotoxic activities

## Abstract

The marine fungus *Chondrostereum* sp. was collected from a soft coral of the species *Sarcophyton tortuosum* from the South China Sea. Three new compounds, chondrosterins F–H (**1**, **4** and **5**), together with three known compounds, incarnal (**2**), arthrosporone (**3**), and (2*E*)-decene-4,6,8-triyn-1-ol (**6**), were isolated. Their structures were elucidated primarily based on NMR and MS data. Incarnal (**2**) exhibited potent cytotoxic activity against various cancer cell lines.

## 1. Introduction

**Figure 1:**
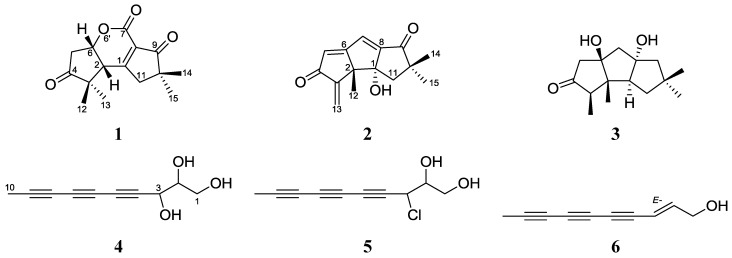
Chemical structures of compounds **1**–**6**.

The marine fungus *Chondrostereum* sp. nov. (collection No. SF002) was collected from the inner tissue of a soft coral of the species *Sarcophyton tortuosum.* The previous analysis of metabolites afforded new hirsutane sesquiterpenoids chondrosterins A–E and hirsutanol E, together with the known compounds hirsutanols A, C and F (gloeosteretriol) [1,2]. Hirsutanol A and chondrosterin A were found to have potent cytotoxic effects on various cancer cell lines and can induce autophagic cell death by increasing reactive oxygen species production [1,3,4]. During our continuing research on the metabolites of *Chondrostereum* sp., a new hirsutane sesquiterpenoid derivate, chondrosterin F (**1**); two new linear polyacetylenes, chondrosterins G–H (**4** and **5**); and three known compounds, incarnal (**2**), arthrosporone (**3**), and (2*E*)-decene-4,6,8-triyn-1-ol (**6**) (Figure 1), were isolated. The structures of these compounds were elucidated primarily based on NMR and MS data. Incarnal (**2**) exhibited significant cytotoxic activities against various cancer lines. Herein we describe the isolation, structural elucidation, and biological evaluation of these compounds.

## 2. Results and Discussion

**Figure 2:**
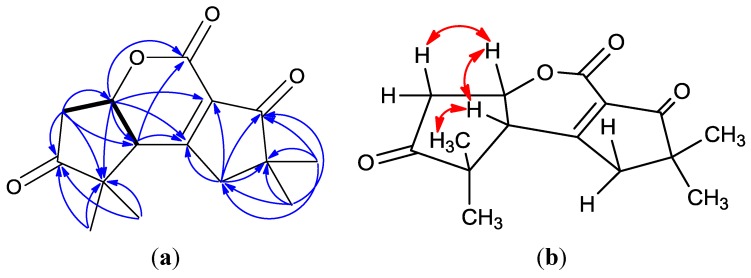
(**a**) ^1^H–^1^H COSY correlations (bold lines), the main HMBC correlations (arrows); and (**b**) key ROESY correlations for **1**.

Chondrosterin F (**1**) was obtained as a colorless oil. The molecular formula of **1** was established as C_15_H_18_O_4 _based on the HREIMS peak at *m*/*z* 262.1202 [M]^+^ and the ^13^C NMR data (Table 1). The strong IR absorptions at 1775 and 1705 cm^−1^ indicated the presence of unconjugated and conjugated carbonyl groups, respectively. The ^13^C and DEPT NMR spectra displayed four methyls, two methylenes, two methines, and seven quaternary carbons. The two carbonyl carbons (δ_C_ 206.5 and 206.0), one ester group (δ_C_ 174.7), and one tetrasubstituted double bond (δ_C_ 186.7 and 142.5) accounted for four degrees of unsaturation. Thus, **1** must be tricyclic to account for the seven degrees of unsaturation required by the molecular formula. The cross-peaks of H-5α/H-6, H-5β/H-6, and H-6/H-2 in the ^1^H–^1^H COSY spectrum indicated the presence of a –CH_2_–CH–CH– structure in this molecule. The two methyl singlets at δ_H_ 1.49 and 2.21 correspond to a geminal pair connected to quaternary carbon C-3 (δ_C_ 62.2), and the other two methyl singlets at δ_H_ 1.16 and 1.24 were connected to quaternary carbon C-10 (δ_C_ 51.0) according to their HMBC correlations (Figure 2a). The HMBC correlations of H-2/C-3, H-5/C-3, H-5/C-4, H-12/C-4 and H-13/C-4 revealed the presence of a substituted five-membered ring. The HMBC correlations between H-11 and C-1, C-8, C-9, and C-10 established the other five-membered ring. The presence of three partial structures—two five-membered rings and an ester group—established the planar structure of compound **1**. The ROESY correlations of H-2/H-6, H-2/H-12, and H-5β/H-6 (Figure 2b) confirmed that all these protons were β-oriented. Compound **1** has a novel rearranged hirsutane skeleton, which could be derived by the migration of a methyl group from C-2 to C-3 and the conversion of the cyclic ketone into a lactone by the Baeyer-Villiger monooxygenase.

**Table 1:** ^1^H and ^13^C NMR data for compounds **1**–**3** obtained at 500/125 MHz, respectively, in CDCl_3_.

Position	1		2		3	
	δ_C_, type	δ_H_, mult., ( *J*)	δ_C_, type	δ_H_, mult., ( *J*)	δ_C_, type	δ_H_, mult., ( *J*)
1	186.7, C		83.6, C		60.5, CH	2.54, dd (12.0, 8.0)
2	79.0, CH	5.57, ddd (7.0, 3.0, 1.0)	63.2, C		54.5, C	
3	62.2, C		147.5, C		55.5, CH	2.56, dd (7.0, 1.0)
4	206.5, C		195.3, C		216.5, C	
5	31.3, CH_2_	α: 2.72, dd (18.0, 10.0);	128.4, CH	6.39, s	49.8, CH_2_	α: 2.19, d (19.0);
		β: 2.28, dd (18.0, 7.5)				β: 2.66, dd (19.0, 1.0)
6	52.2, CH	3.43, ddd (10.0, 7.5, 7.0)	184.3, C		87.0, C	
7	174.7, C		126.5, CH	7.18, s	56.8, CH_2_	α: 2.38, d (15.0);
						β: 2.19, d (15.0)
8	142.5, C		157.3, C		90.9, C	
9	206.0, C		207.3, C		58.8, CH_2_	α: 1.94, d (14.0);
						β: 1.77, dd (14.0, 3.0)
10	51.0, C		51.1, C		40.1, C	
11	40.5, CH_2_	α: 2.38, dd (19.0, 3.0);	42.5, CH_2_	2.24, d (14.0);	44.7, CH_2_	α: 1.69, dd (12.0, 12.0);
		β: 2.75, dd (19.0, 1.0)		2.04, d (14.0)		β: 1.58, ddd (12.0, 8.0, 3.0)
12	23.5, CH_3_	1.49, s	26.4, CH_3_	1.27, s	11.3, CH_3_	0.83, s
13	27.6, CH_3_	2.21, s	116.6, CH_2_	6.18, s;	8.2, CH_3_	1.02, d (7.0)
				5.39, s		
14	25.0, CH_3_	1.16, s	27.3, CH_3_	1.40, s	26.8, CH_3_	1.13, s
15	25.3, CH_3_	1.24, s	25.9, CH_3_	1.21, s	29.6, CH_3_	1.07, s
1α-OH				2.05, brs		
6β-OH						1.54, s
8α-OH						1.74, s

Compound **2** was identified as incarnal, which was first isolated by Hideki Takazawa and co-workers from the fungus *Gloeostereum incarnatum* [5]. Our NMR spectra recorded in CDCl_3_ (see Table 1) were slightly different from the reference spectra. Through comprehensive analysis of the 1D (^1^H and ^13^C) and 2D NMR (gHMQC, HMBC, ^1^H–^1^H COSY, and ROESY) spectra, the two carbonyl groups at C-4 and C-9 were assigned to δ_C_ 195.3 and 207.3, respectively. The ^13^C and ^1^H NMR signals of three methyl groups were assigned as follows: CH_3_-12: δ_C_ 26.4, δ_H_ 1.27, s; CH_3_-14: δ_C_ 27.3, δ_H_ 1.40, s; and CH_3_-15: δ_C_ 25.9, δ_H_ 1.21, s, respectively. The observed *J* value for the coupling between H-11α and H-11β is 14.0 Hz, whereas the reported reference value is 5.1 Hz [5], which is surprisingly lower than the typical value for this type of coupling. 

Compound **3**, identified as arthrosporone, has been found in an unidentified arthroconidial fungus [6] and in *Macrocystidia cucumis* [7]. Careful analysis of our data indicated that these data are consistent with the data reported by Steglich *et al.* [7].

Chondrosterin G (**4**) was obtained as a colorless solid. The molecular formula was established as C_10_H_10_O_3_ based on the EIMS peak at *m*/*z* 178 [M]^+^ and the NMR data (Table 2). The ^13^C and DEPT NMR spectra contained signals for one methyl, one methylene, two methines, and six quaternary carbons. HMBC correlations revealed that the three hydroxyl groups at δ_H_ 4.53 (t, *J* = 5.4 Hz), 5.01 (d, *J* = 5.5 Hz), and 5.68 (d, *J* = 6.5 Hz) are connected to CH_2_ (δ_C_ 62.2; δ_H_ 3.41, ddd, *J* = 11.0, 11.0, 5.5 Hz; 3.37, ddd, *J* = 11.0, 5.5, 5.5 Hz), CH (δ_C_ 74.1; δ_H_ 3.46, ddd, *J* = 10.0, 5.0, 5.0 Hz), and CH (δ_C_ 63.2; δ_H_ 4.29, t, *J* = 6.0 Hz) moieties, respectively. These units formed the structural triol fragment –CH(OH)–CH(OH)–CH_2_OH. The methyl group (δ_H_ 2.03, s) was connected to a quaternary carbon, and the small chemical shift of this carbon (δ_C_ 3.8) indicated that it was located in a strongly shielded environment. The six quaternary carbons generate the six degrees of unsaturation required by the molecular formula. Thus, the linear polyacetylene skeleton was readily established. Finally, the structure of compound **4** was determined to be deca-4,6,8-triyn-1,2,3-triol.

**Table 2:** ^13^C NMR data for compounds **4**–**6**.

Position	4 ^a^, δ_C_, type	5 ^b^, δ_C_, type	5 ^c^, δ_C_, type	6 ^c^, δ_C_, type
1	62.2, CH_2_	62.6, CH_2_	62.8, CH_2_	62.7, CH_2_
2	74.1, CH	74.6, CH	74.6, CH	146.8, CH
3	63.2, CH	51.1, CH	49.9, CH	108.4, CH
4–9 ^d^	79.6, C	80.2, C	78.3, C	78.2, C
	78.4, C	73.4, C	73.4, C	75.2, C
	68.3, C	71.0, C	70.6, C	73.6, C
	63.8, C	64.9, C	66.3, C	67.2, C
	62.5, C	63.7, C	64.6, C	64.9, C
	58.7, C	57.8, C	57.8, C	59.0, C
10	3.8, CH_3_	4.0, CH_3_	4.8, CH_3_	4.6, CH_3_

^a^ Measured in DMSO-*d*_6_ (125 MHz); ^b^ Measured in DMSO-*d*_6_ (100 MHz); ^c^ Measured in CDCl_3_ (125 MHz); ^d^ These data may be interchanged.

Chondrosterin H (**5**) was obtained as a white solid. The molecular formula was established as C_10_H_9_O_2_Cl based on the APCI-(+) MS and NMR data (Table 3). The ^13^C and DEPT NMR spectra contained signals for one methyl, one methylene, two methines, and six quaternary carbons, as observed for compound **4**. The two hydroxyl groups at δ_H_ 4.90 (t, *J* = 4.8 Hz) and 5.74 (d, *J* = 6.0 Hz) were connected to C-1 and C-2, respectively, according to the HMBC correlations. Therefore, the Cl was placed at C-3. Based on these assignments, compound **5** was established as 3-chlorodeca-4,6,8-triyn-1,2-diol.

**Table 3:** ^1^H NMR data for compounds **4**–**6** (δ, mult., *J* in Hz).

Position	4 ^a^	5 ^b^	5 ^c^	6 ^c^
1	3.41, ddd (11.0, 11.0, 5.5);	3.45, ddd (11.2, 6.0, 4.8);	3.85, d (4.5)	4.25, dd (4.5, 2.0)
	3.37, ddd (11.0, 5.5, 5.5)	3.39, ddd (11.2, 6.0, 4.8)		
2	3.46, ddd (10.0, 5.0, 5.0)	3.70, dddd (6.0, 6.0, 6.0, 4.0)	3.95, dt (6.0, 4.5)	6.46, dt (16.0, 4.5)
3	4.29, t (6.0)	4.99, d (4.0)	4.74, d (6.0)	5.84, dt (16.0, 2.0)
10	2.03, s	2.04, s	1.99, s	1.98, s
1-OH	4.53, t (5.4)	4.90, t (4.8)	1.91, brs	1.51, brs
2-OH	5.01, d (5.5)	5.74, d (6.0)	1.91, brs	
3-OH	5.68, d (6.5)			

^a^ Measured in DMSO-*d*_6_ (500 MHz); ^b^ Measured in DMSO-*d*_6_ (400 MHz); ^c^ Measured in CDCl_3_ (500 MHz).

Compound **6** was identified as (2*E*)-decene-4,6,8-triyn-1-ol (dehydromatricarianol), which is a common polyacetylene metabolite produced by basidiomycete fungi [8]. The *J* value for the coupling between H-2 and H-3 was 16.0 Hz, confirming that the double bond had the *E*-configuration.

Compounds **4**–**6** can be slowly oxidized in the air. When heated, this oxidation can be accelerated. Compound **6**was the primary polyacetylenic metabolite isolated from the culture and is believed to be the biosynthetic precursor of several fungal polyacetylenes [9]. Therefore, we hypothesized that **6** is the biosynthetic precursor of **4** and **5**. The epoxidation of the double bond of **6** followed by hydrolysis gives a diol (**4**), whereas Cl^−^, acting as a nucleophile and working together with H^+^, reacts with the epoxide to afford the halogenated alcohol (**5**).

Eight cancer cell lines were used to examine the cytotoxic activities of compounds **1**–**3** *in vitro*. This assay revealed that **2** had potent cytotoxic effects on these cancer cell lines (Table 4). In contrast, **1** and **3** were apparently inactive in this assay (IC_50_ values >50 μg/mL). 

**Table 4:** Cytotoxic activitiesof **2** *in vitro*.

Cancer cell line	IC_50_ (μg/mL)
Human nasopharyngeal carcinoma cell line CNE1	8.33
Human nasopharyngeal carcinoma cell line CNE2	6.07
Human nasopharyngeal carcinoma cell line SUNE1	3.99
Human lung cancer cell line A549	12.37
Human colon cancer cell line Lovo	2.16
Human epidermoid carcinoma cell line KB	28.55
Human hepatic cancer cell line Bel7402	23.36
Human breast cancer cell line MCF-7	4.57

## 3. Experimental Section

### 3.1. General Experimental Procedures

Preparative HPLC was performed using a Shimadzu LC-20AT HPLC pump equipped with an SPD-20A dual λ absorbance detector and a Shim-pack PRC-ODS HPLC column (250 × 20 mm). Melting points were determined using an X-6 micro-melting point apparatus (Beijing Fukai Science and Technology Development, Beijing, China) and are uncorrected. Optical rotations were measured using a Schmidt and Haensch Polartronic HNQW5 optical rotation spectrometer. IR spectra were recorded on a Nicolet Avatar 330 FT-IR spectrophotometer. UV spectra were recorded on a Shimadzu UV-Vis-NIR spectrophotometer. 1D and 2D NMR spectra were recorded on a Varian Inova 500 spectrometer and a Bruker Avance II 400 spectrometer. The chemical shifts are relative to the residual solvent signals (CDCl_3_: δ_H_ 7.26 and δ_C_ 77.0; DMSO-*d*_6_: δ_H_ 2.50 and δ_C_ 39.51). HPLC-MS analyses were performed using a Thermo Finnigan LCQ™ DECA XP liquid chromatography-mass spectrometry system. Mass spectra were obtained on a Thermo DSQ EI low-resolution mass spectrometer and a Thermo MAT95XP EI high-resolution mass spectrometer. 

### 3.2. Fungal Strain and Culture Method

*Chondrostereum* sp. nov. (collection No. SF002) was isolated from the inner tissue of a soft coral of the species *Sarcophyton tortuosum* collected from the Hainan Sanya National Coral Reef Reserve, China. This fungal strain was maintained on potato dextrose agar (PDA) slants. The fermentation medium was potato dextrose broth (PDB, potatoes 200 g, dextrose 20 g, seawater 1 L). Plugs of agar supporting mycelium growth were cut and transferred aseptically to a 500 mL Erlenmeyer flask containing 200 mL of liquid PDB medium. The liquid medium was sterilized at 120 °C for 30 min. The flask was incubated at 28 °C on a rotary shaker (120 rpm) for five days. The mycelia were aseptically transferred to 500 mL Erlenmeyer flasks containing 200 mL of the same liquid medium. The flasks were then incubated at 28 °C on a rotary shaker (120 rpm) for 20 days. 

### 3.3. Extraction and Isolation

Sixty liters of liquid culture was filtered through cheesecloth. The culture broth was successively extracted three times with EtOAc. The EtOAc extract was concentrated by low-temperature rotary evaporation. The extract (24.6 g) was chromatographed on a silica gel column using petroleum ether-EtOAc (100:0–0:100) followed by EtOAc-MeOH (100:0–0:100) as the eluent to afford 18 fractions (code Fr. 1–Fr. 18). Fr. 12 was further purified by RP-HPLC with a gradient of H_2_O-MeCN (40:60 up to 0:100, v/v) to afford compounds **1** (2 mg), **2** (9 mg), and **3** (18 mg). Fr. 7–8 was further purified by Sephadex LH-20 gel column chromatography eluted with CHCl_3_-MeOH (2:1, v/v) and by repeated RP-HPLC eluted with H_2_O-MeCN (60:40, v/v) to yield compounds **4** (12 mg), **5** (15 mg), and **6** (36 mg).

Chondrosterin F (**1**): Colorless oil; [α]^20^_D_ +149 (*c* 0.032, MeOH); UV (MeOH) λ_max_ (ε) 233 nm (6780); IR (KBr) υ_max_ 2963, 2927, 2868, 1775, 1705, 1651, 1462, 1423, 1398, 1360, 1226, 1178, 1116, 1031, 977 cm^−^^1^; ^1^H and ^13^C NMR data, see Table 1; LREIMS *m*/*z* 262, 247, 220, 205, 177, 164, 159, 150, 131, 119, 105, 77, 65, 55; HREIMS *m*/*z* 262.1202 [M]^+^ (calcd for C_15_H_18_O_4_, 262.1200). 

Incarnal (**2**): Red solid; IR (KBr) υ_max_ 3417, 2965, 2931, 2868, 1691, 1644, 1597, 1457, 1380, 1260, 1209, 1166, 1126, 1061, 1030, 998, 953, 898, 864, 629 cm^−1^; ^1^H and ^13^C NMR data, see Table 1; LREIMS *m*/*z* 244, 229, 216, 201, 188, 183, 173, 160, 155, 145, 132, 115, 104, 91, 77, 69, 63, 55, 51. 

Arthrosporone (**3**): White solid; [α]^20^_D_ −49.8 (*c* 0.034, MeOH). UV (MeOH) λ_max_ (ε): 228 nm (123), 206 nm (154). IR (KBr) υ_max_ 3545, 3399, 3312, 2982, 2944, 2867, 1721, 1459, 1403, 1375, 1311, 1262, 1183, 1138, 1117, 1086, 1057, 1018, 1000, 937, 890, 854 cm^−1^; ^1^H and ^13^C NMR data, see Table 1; LREIMS *m*/*z* 252, 234, 219, 201, 192, 177, 173, 163, 159, 150, 135, 125, 110, 95, 91, 83, 79, 69, 55.

Chondrosterin G (**4**): White solid; IR (KBr) υ_max_ 3412, 2928, 1696, 1647, 1430, 1388, 1205, 1110, 1077, 1044, 1000 cm^−1^; ^1^H and ^13^C NMR data, see Table 2. LREIMS *m*/*z* 178, 160, 131, 118, 100, 89, 74, 63, 51.

Chondrosterin H (**5**): White solid; IR (KBr) υ_max_ 3410, 1684, 1458, 1451, 1209, 1145, 849, 801, 726, 604 cm^−1^; ^1^H and ^13^C NMR data, see Table 2. APCI-(+) MS *m*/*z* 219 [M + Na]^+^, 197 [M + H]^+^, 179 [M + H − H_2_O]^+^, 161 [M + H − HCl]^+^, 149, 128, 119, 105, 94, 80, 74, 62.

(2*E*)-Decene-4,6,8-triyn-1-ol (dehydromatricarianol, **6**): White solid; IR (KBr) υ_max_ 3433, 2926, 1676, 1438, 1400, 1381, 1237, 1067, 879 cm^−1^. ^1^H and ^13^C NMR data, see Table 2.

### 3.4. Cytotoxicity Assay

The *in vitro* cytotoxicities of **1**–**3** were determined using the colorimetric MTT (3-(4,5-dimethylthiazol-2-yl)-2,5-diphenyl-2*H*-tetrazolium bromide) assay. The tested human cancer cell lines were seeded in 96-well plates at a density of 3 × 10^7^ cells/L, and the compounds were added at various concentrations (0.125–50 mg/L). After 48 h, MTT was added to the culture medium at a final concentration of 0.5 mg/mL, and the plates were incubated for 4 h at 37 °C. The supernatant was removed. The formazan crystals were dissolved in DMSO (150 μL) with gentle shaking at r.t. The absorbance at 570 nm was recorded with a microplate reader (Bio-Rad, Hercules, California, CA, USA), and the data were analyzed with the SPSS 13.0 software package.

## 4. Conclusions

Compounds **1**–**3** are hirsutane sesquiterpenoids; however, compounds **4**–**6** are polyacetylene derivatives. Our results indicate that the marine fungus *Chondrostereum* sp. can produce novel metabolites with various structures. Hopefully, the systematical analysis of the metabolites of *Chondrostereum* sp. can achieve the goal of “one strain-many compounds (OSMAC)”. Incarnal (**2**) exhibited potent cytotoxic activities. These results also provide additional evidence indicating that the α-methylene ketone functional group is essential to the cytotoxic activities of hirsutane sesquiterpenoids.

## Supplementary Files

Supplementary File 1 Supplementary Information (PDF, 3702 KB)
