# Supplementary material for: Isolation and Structural Elucidation of Chondrosterins F–H from the Marine Fungus Chondrostereum sp
Source: Mar Drugs. 2013 Feb 22;11(2):551–8. doi: 10.3390/md11020551 (PMC3640397; doi:10.3390/md11020551)

Supplementary Materials

Figure S1. HREIMS of chondrosterin F (**1**)
Figure S2. ^{1}H NMR (500 MHz, $CDCl_3$) spectrum of chondrosterin F (**1**)
Figure S3. ^{13}C NMR (125 MHz, $CDCl_3$) spectrum of chondrosterin F (**1**)
Figure S4. gHMQC of chondrosterin F (**1**)
Figure S5. gHMBC of chondrosterin F (**1**)
Figure S6. ^{1}H–^{1}H gCOSY of chondrosterin F (**1**)
Figure S7. ROESY of chondrosterin F (**1**)
Figure S8. ^{1}H NMR (500 MHz, $CDCl_3$) spectrum of incarnal (**2**)
Figure S9. ^{13}C NMR (125 MHz, $CDCl_3$) spectrum of incarnal (**2**)
Figure S10. ^{1}H NMR (500 MHz, $CDCl_3$) spectrum of arthrosporone (**3**)
Figure S11. ^{13}C NMR (125 MHz, $CDCl_3$) spectrum of arthrosporone (**3**)
Figure S12. ^{1}H NMR (500 MHz, DMSO-d_6) spectrum of chondrosterin G (**4**)
Figure S13. ^{13}C NMR (125 MHz, DMSO-d_6) spectrum of chondrosterin G (**4**)
Figure S14. gHMQC spectrum of chondrosterin G (**4**)
Figure S15. gHMBC spectrum of chondrosterin G (**4**)
Figure S16. ^{1}H–^{1}H gCOSY spectrum of chondrosterin G (**4**)
Figure S17. ^{1}H NMR (400 MHz, DMSO-d_6) spectrum of chondrosterin H (**5**)
Figure S18. ^{13}C NMR (100 MHz, DMSO-d_6) spectrum of chondrosterin H (**5**)
Figure S19. ^{1}H NMR (500 MHz, $CDCl_3$) spectrum of chondrosterin H (**5**)
Figure S20. ^{13}C NMR (125 MHz, $CDCl_3$) spectrum of chondrosterin H (**5**)
Figure S21. ^{1}H NMR (500 MHz, $CDCl_3$) spectrum of dehydromatricarianol (**6**)
Figure S22. ^{13}C NMR (125 MHz, $CDCl_3$) spectrum of dehydromatricarianol (**6**)

SPECTRUM-MS

File: D:\DATA-HR\11\121413-sf2-c1.RAW

Full ms [254.100–269.700]-Range: 254.100–264.000

Scan No. 27 of 44

Scan #: 27

RT: 0.98

Data points: 2

Mass	Relative Intensity	Theoretical Mass	Delta [ppm]	Delta [mmu]	RDB	Composition
262.1202	25.2	262.1200	0.9	0.2	7.0	C_{15} H_{18} O_4
263.1276	35.6	263.1278	−0.8	−0.2	6.5	C_{15} H_{19} O_4

Figure S1. HREIMS of chondrosterin F (**1**).

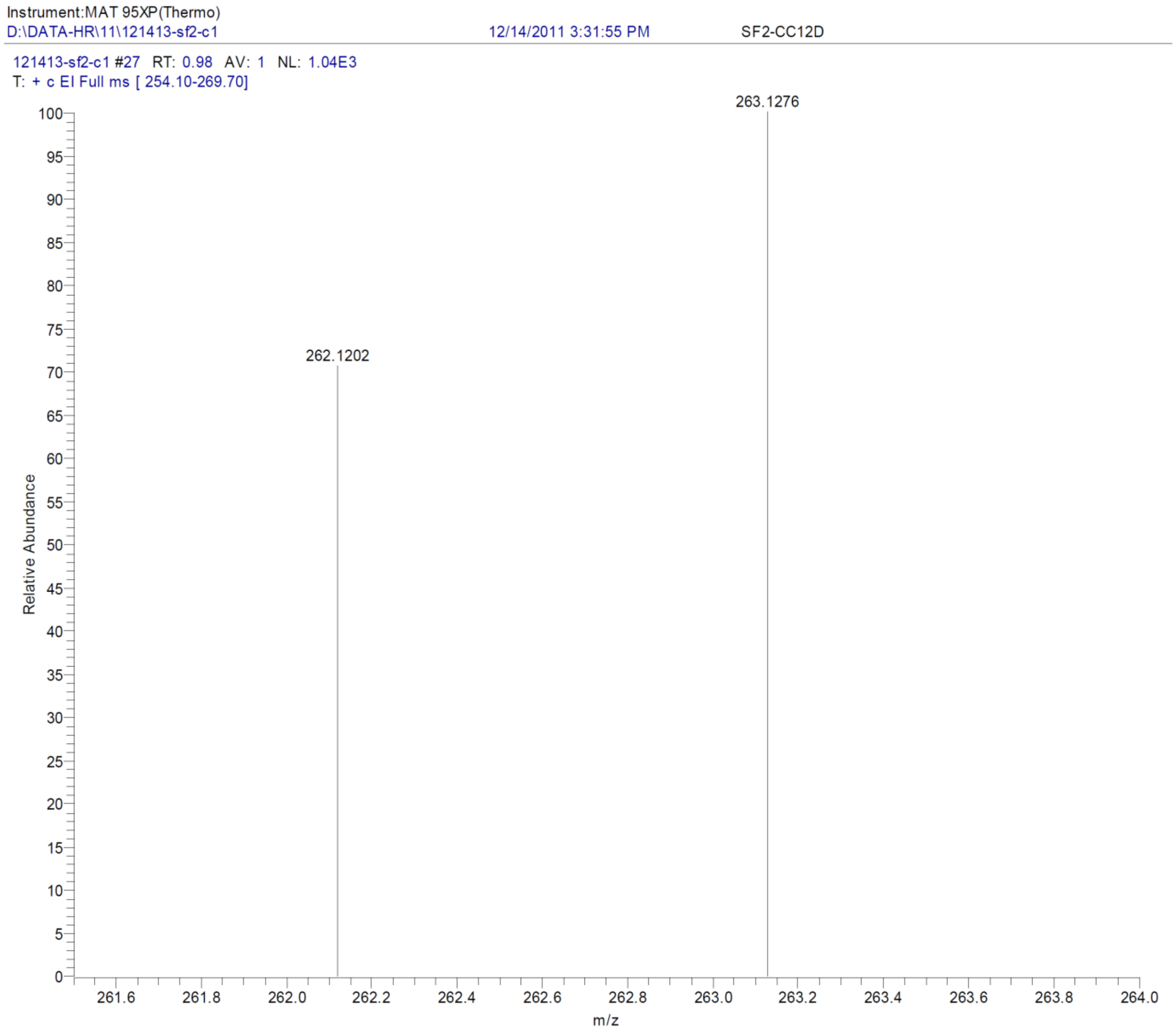

Figure S2. ^{1}H NMR (500 MHz, $CDCl_3$) spectrum of chondrosterin F (**1**).

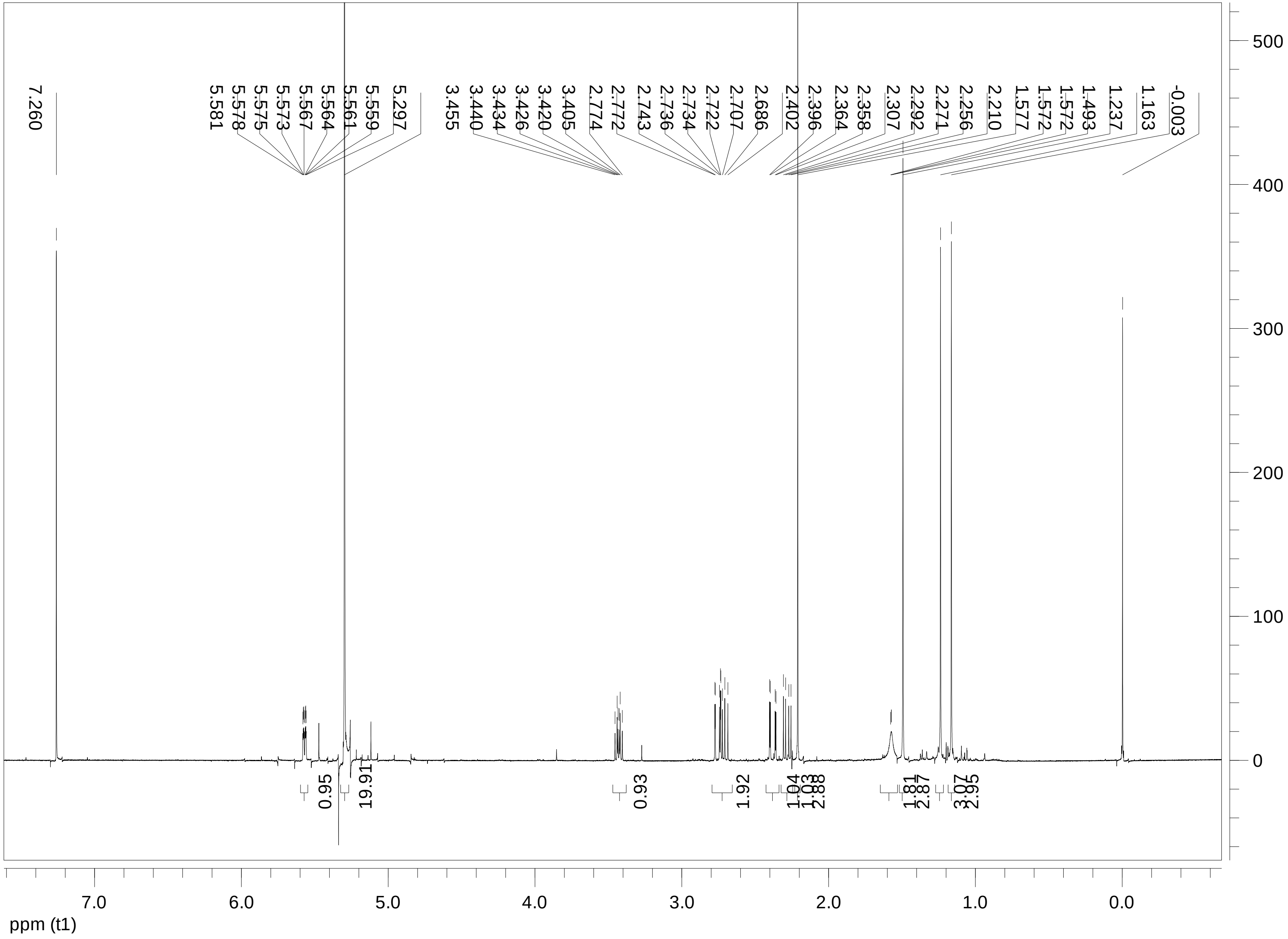

Figure S3. ^{13}C NMR (125 MHz, $CDCl_3$) spectrum of chondrosterin F (**1**).

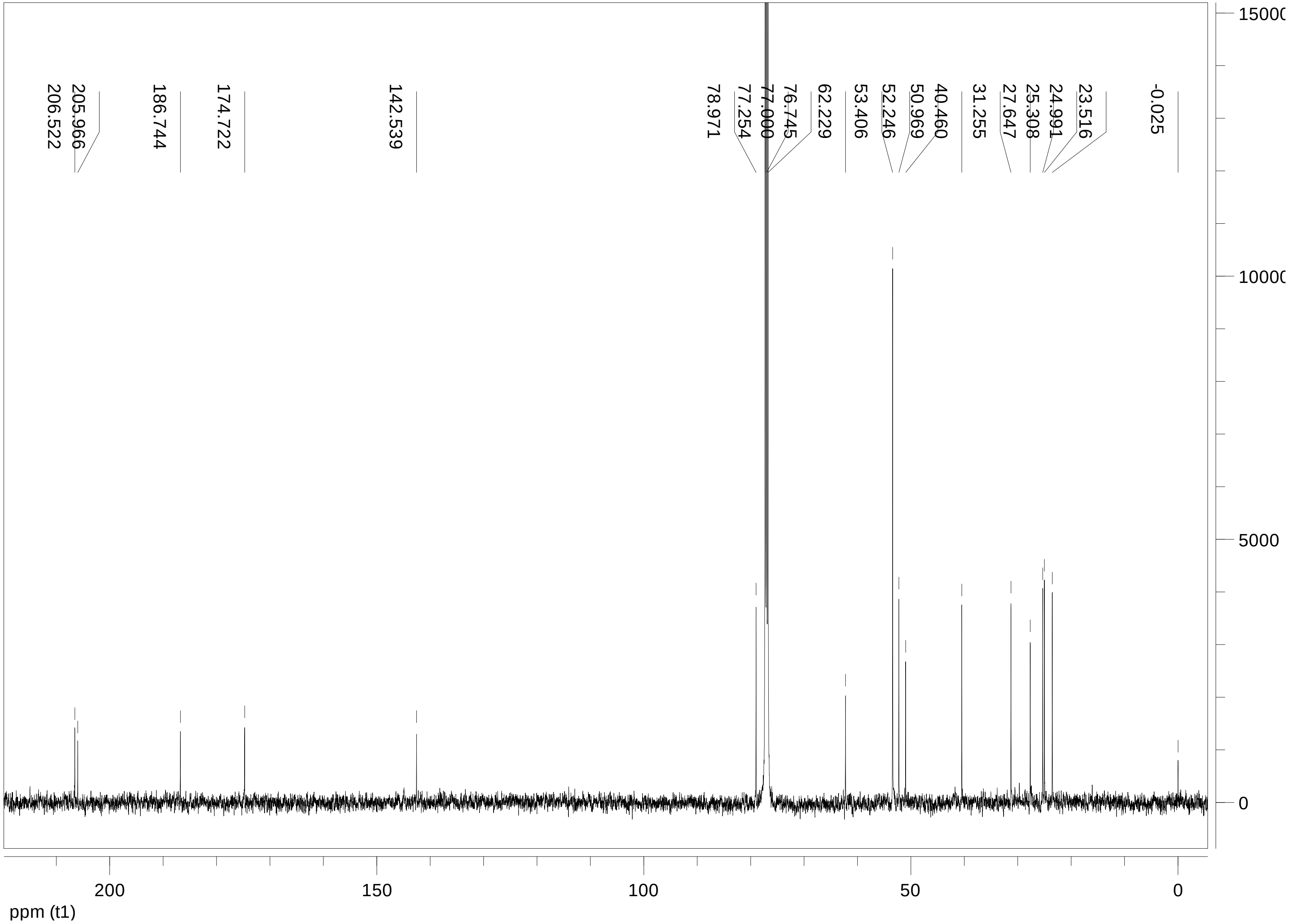

Figure S4. gHMQC of chondrosterin F (**1**).

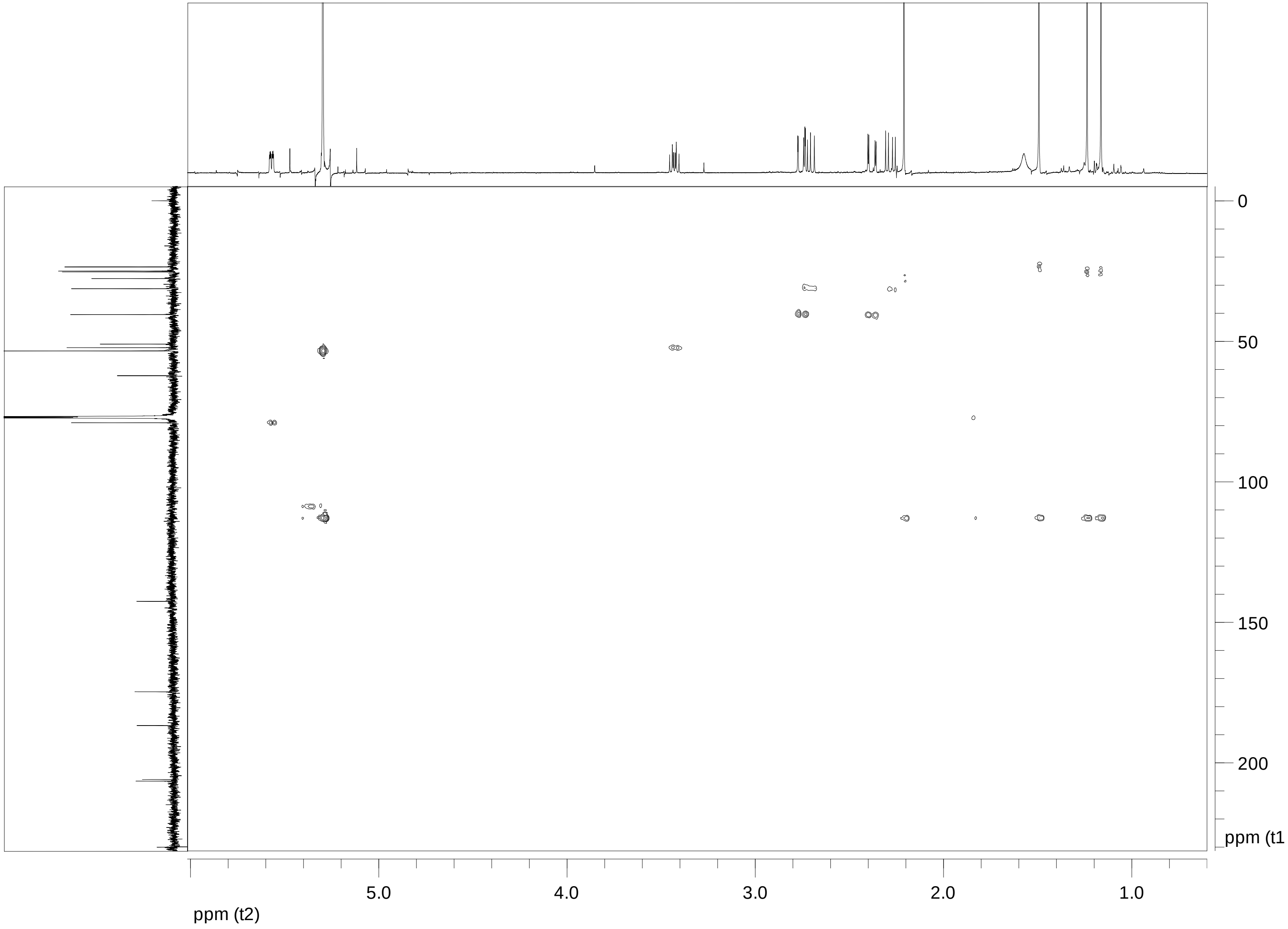

Figure S5. gHMBC of chondrosterin F (**1**).

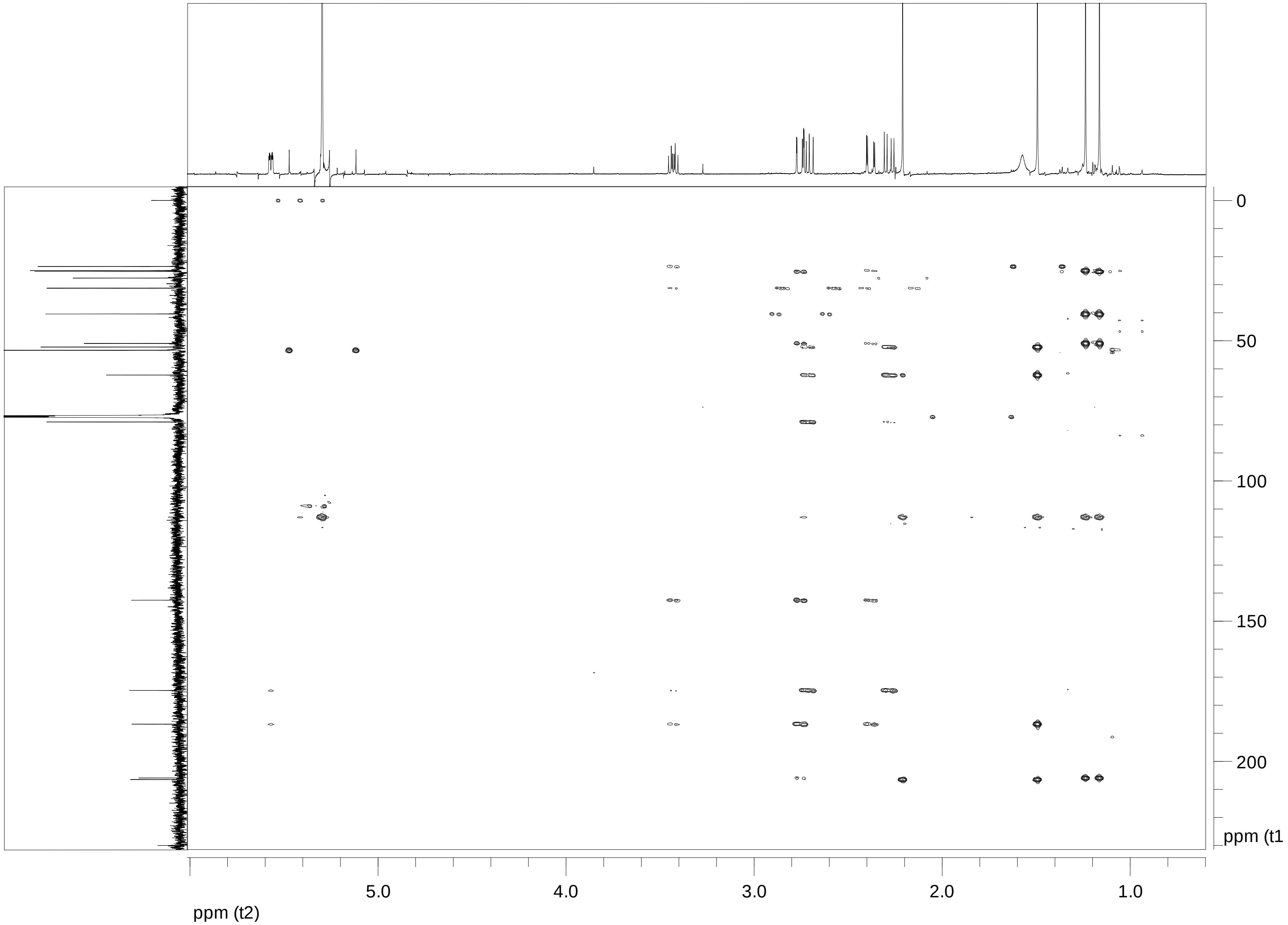

Figure S6. ^{1}H–^{1}H gCOSY of chondrosterin F (**1**).

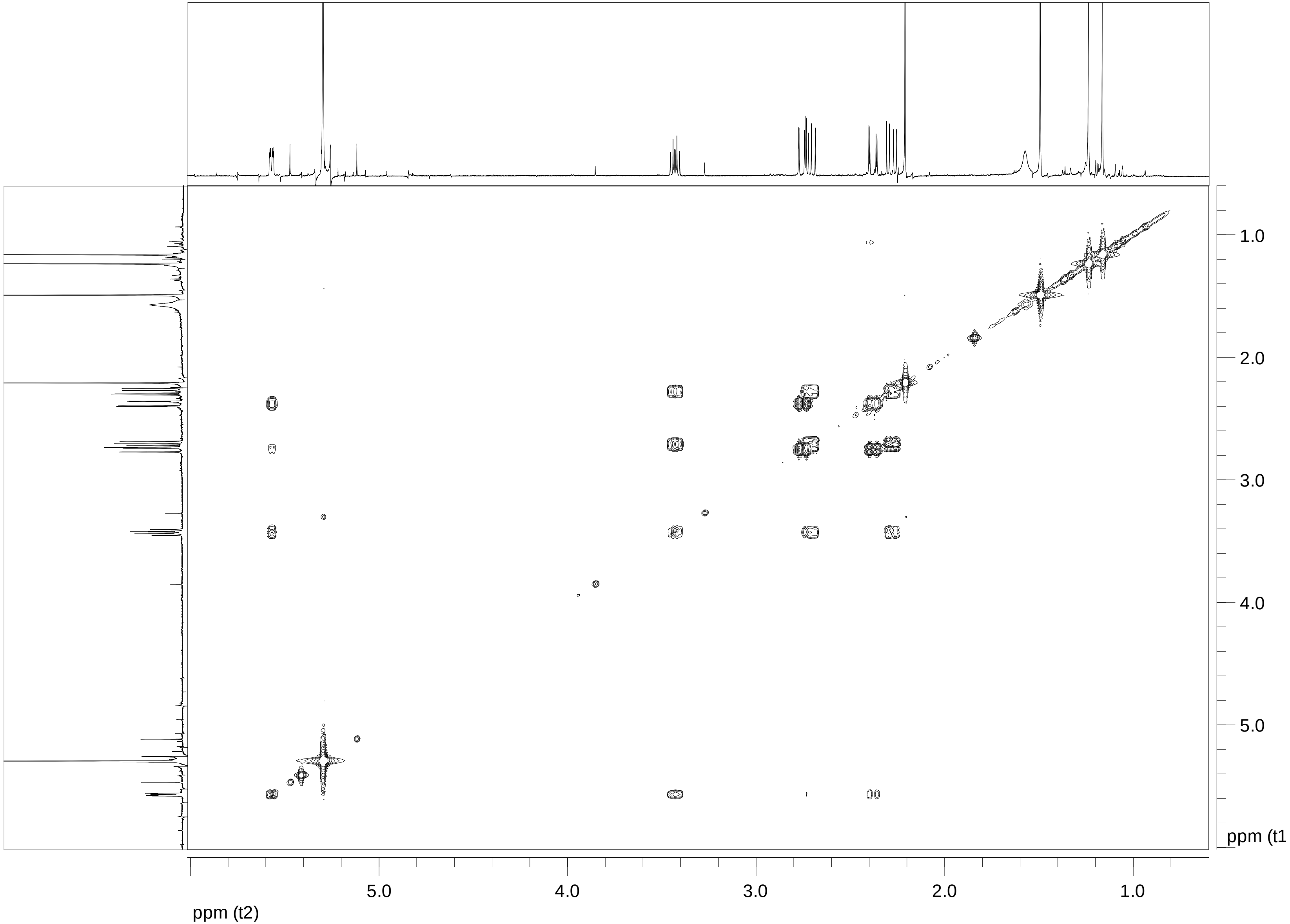

Figure S7. ROESY of chondrosterin F (**1**).

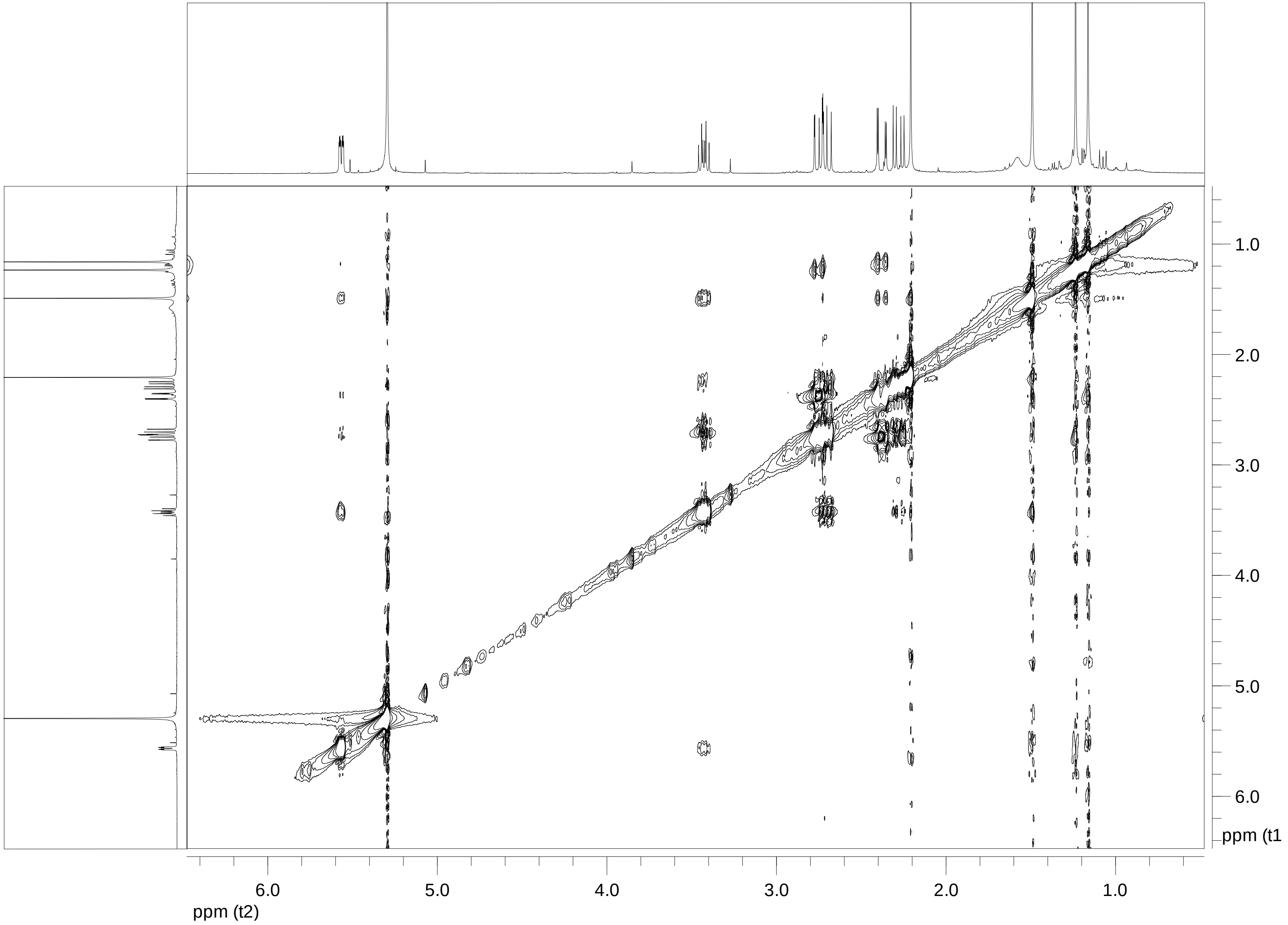

Figure S8. ^{1}H NMR (500 MHz, $CDCl_3$) spectrum of incarnal (**2**).

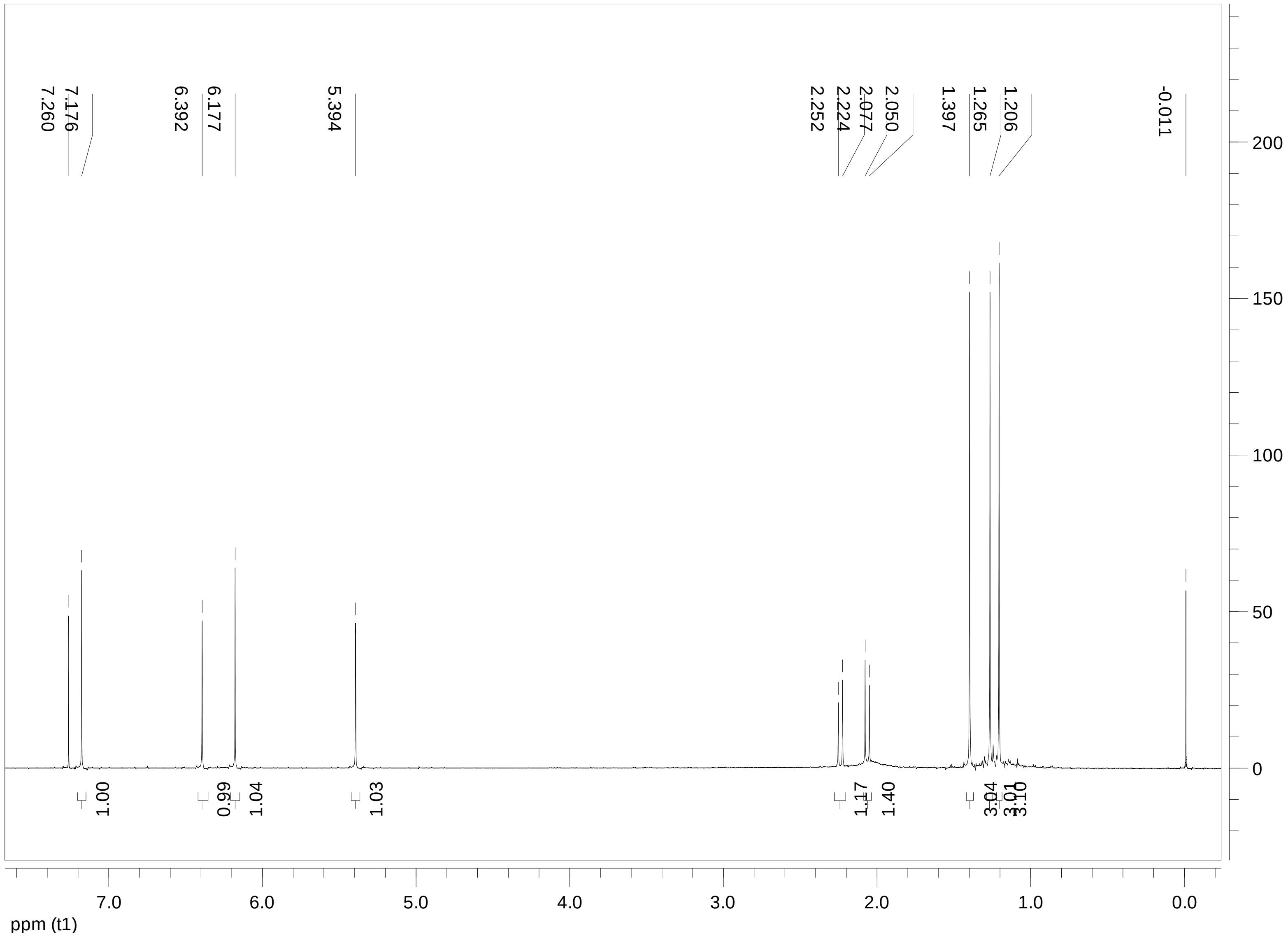

Figure S9. ^{13}C NMR (125 MHz, $CDCl_3$) spectrum of incarnal (**2**).

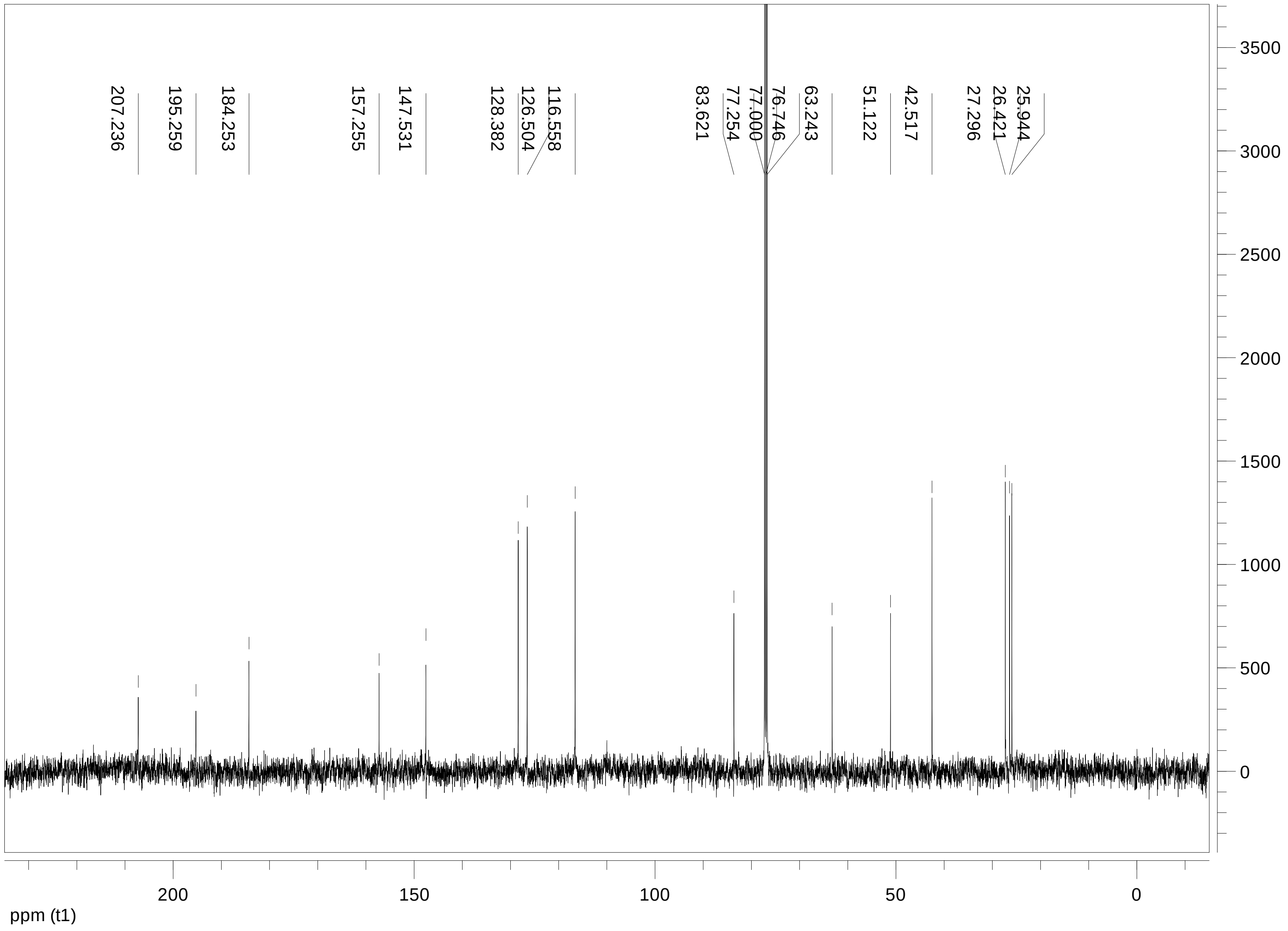

Figure S10. ^1H NMR (500 MHz, $CDCl_3$) spectrum of arthrosporone (**3**).

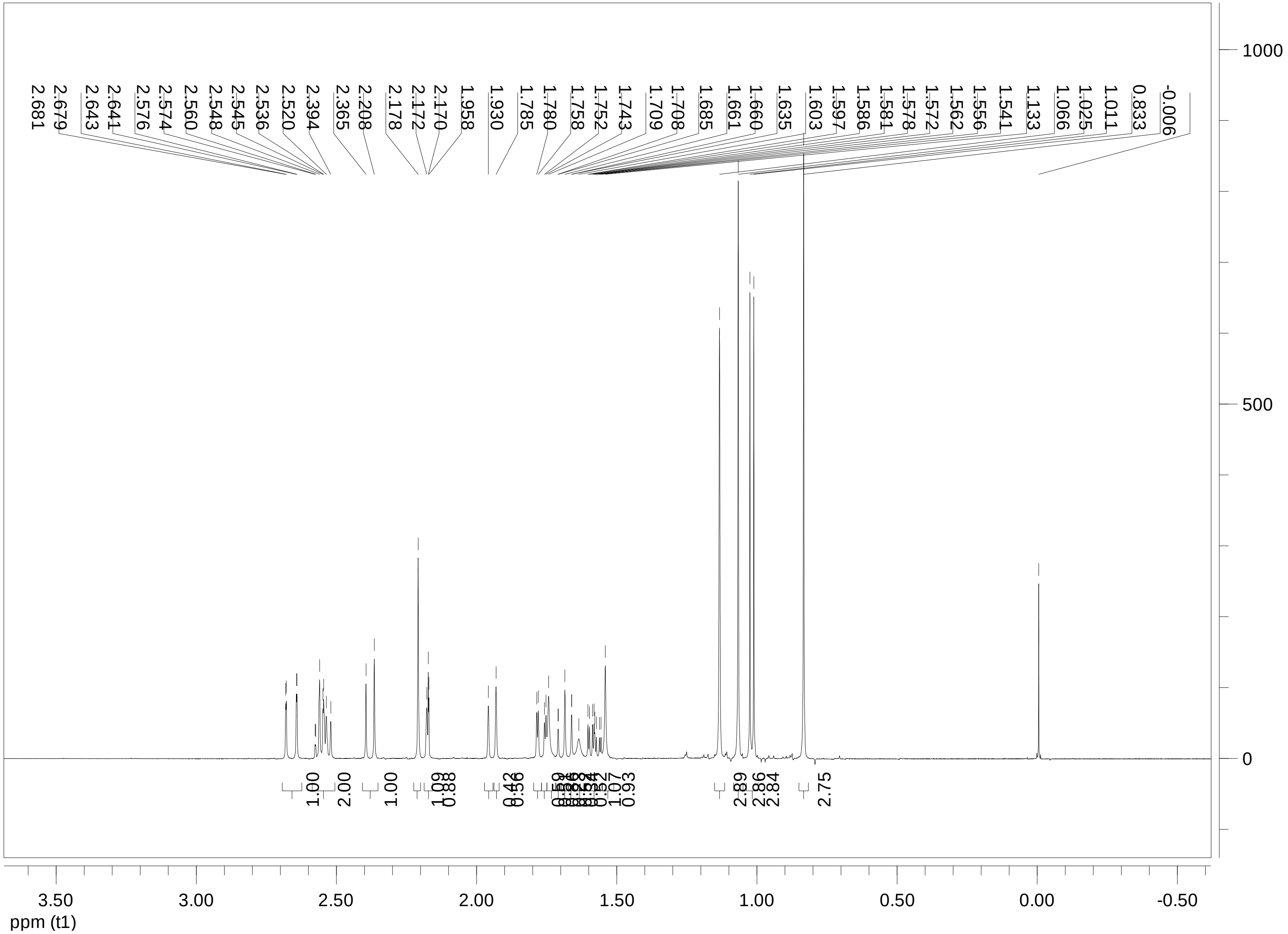

Figure S11. ^{13}C NMR (125 MHz, $CDCl_3$) spectrum of arthrosporone (**3**).

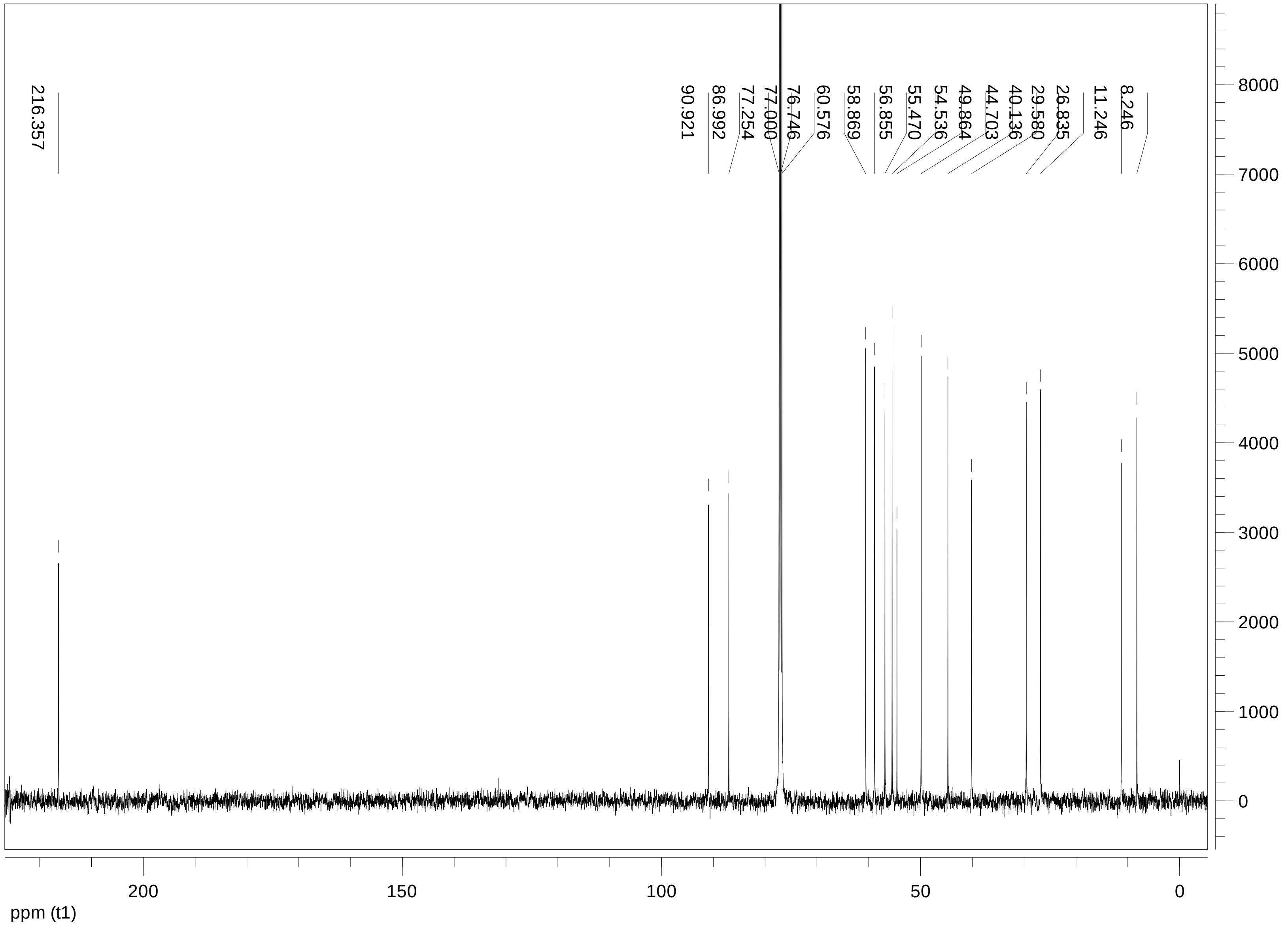

Figure S12. ^{1}H NMR (500 MHz, DMSO-d_6) spectrum of chondrosterin G (**4**).

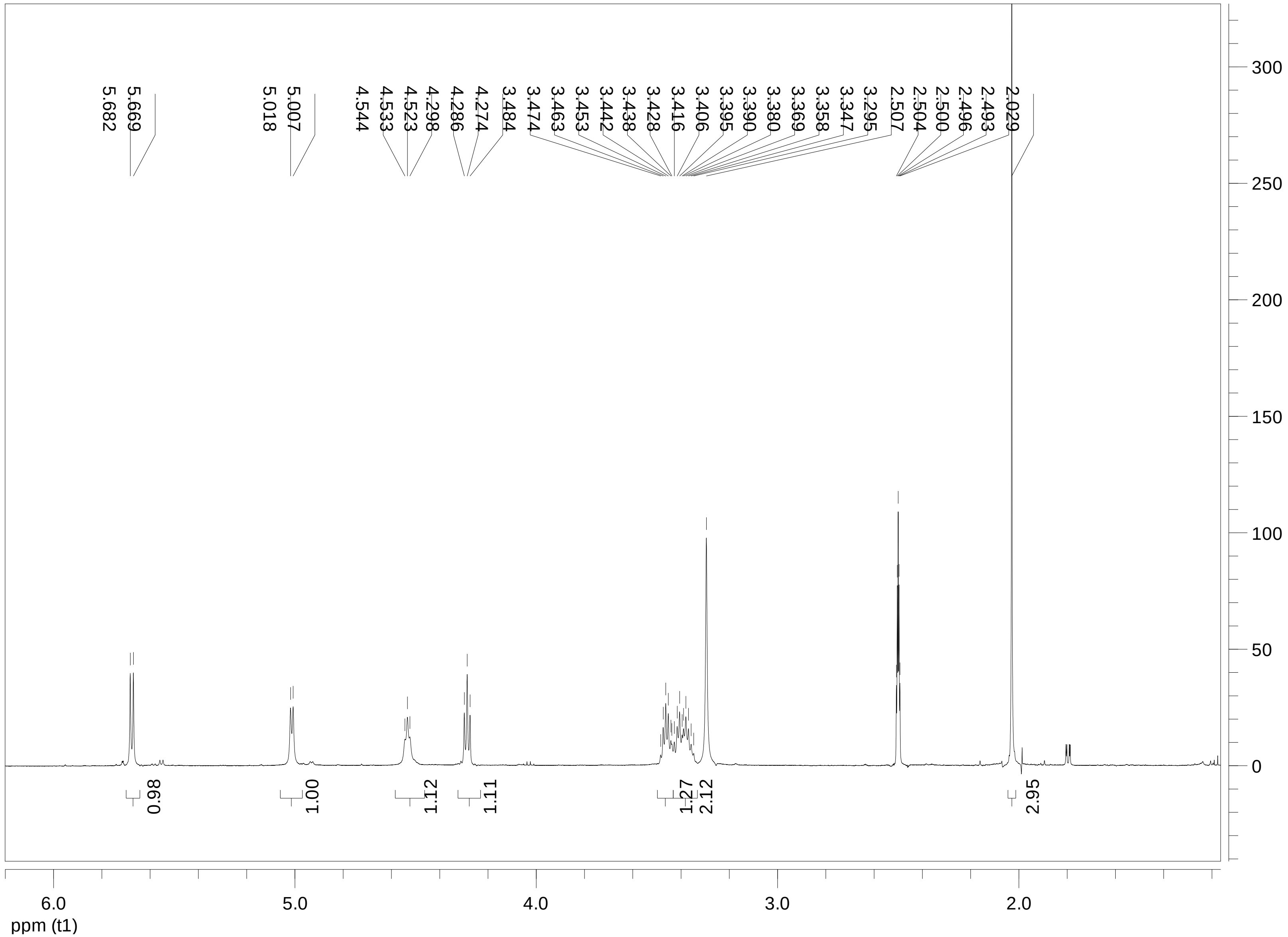

Figure S13. ^{13}C NMR (125 MHz, DMSO-d_6) spectrum of chondrosterin G (**4**).

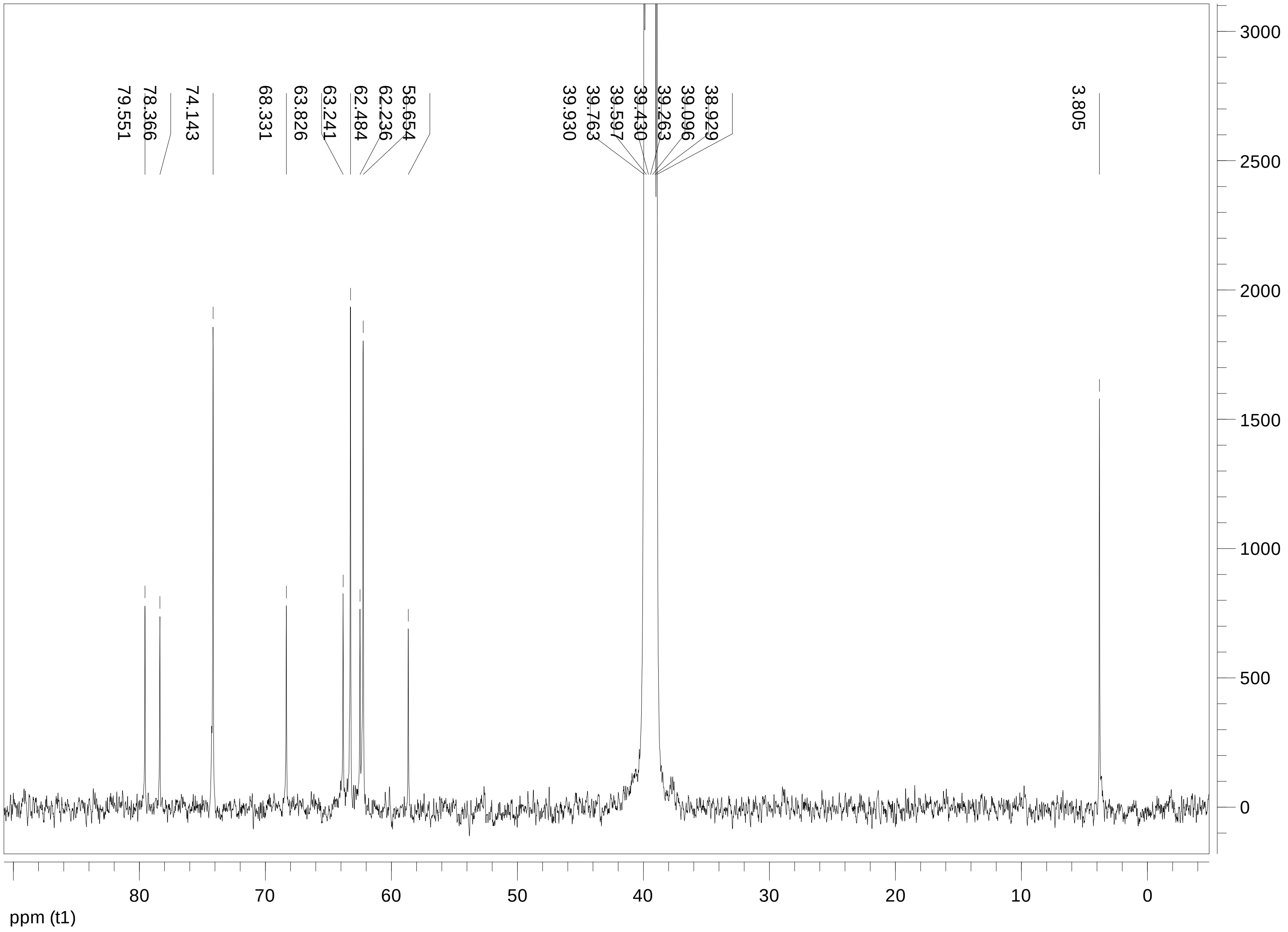

Figure S14. gHMQC spectrum of chondrosterin G (**4**).

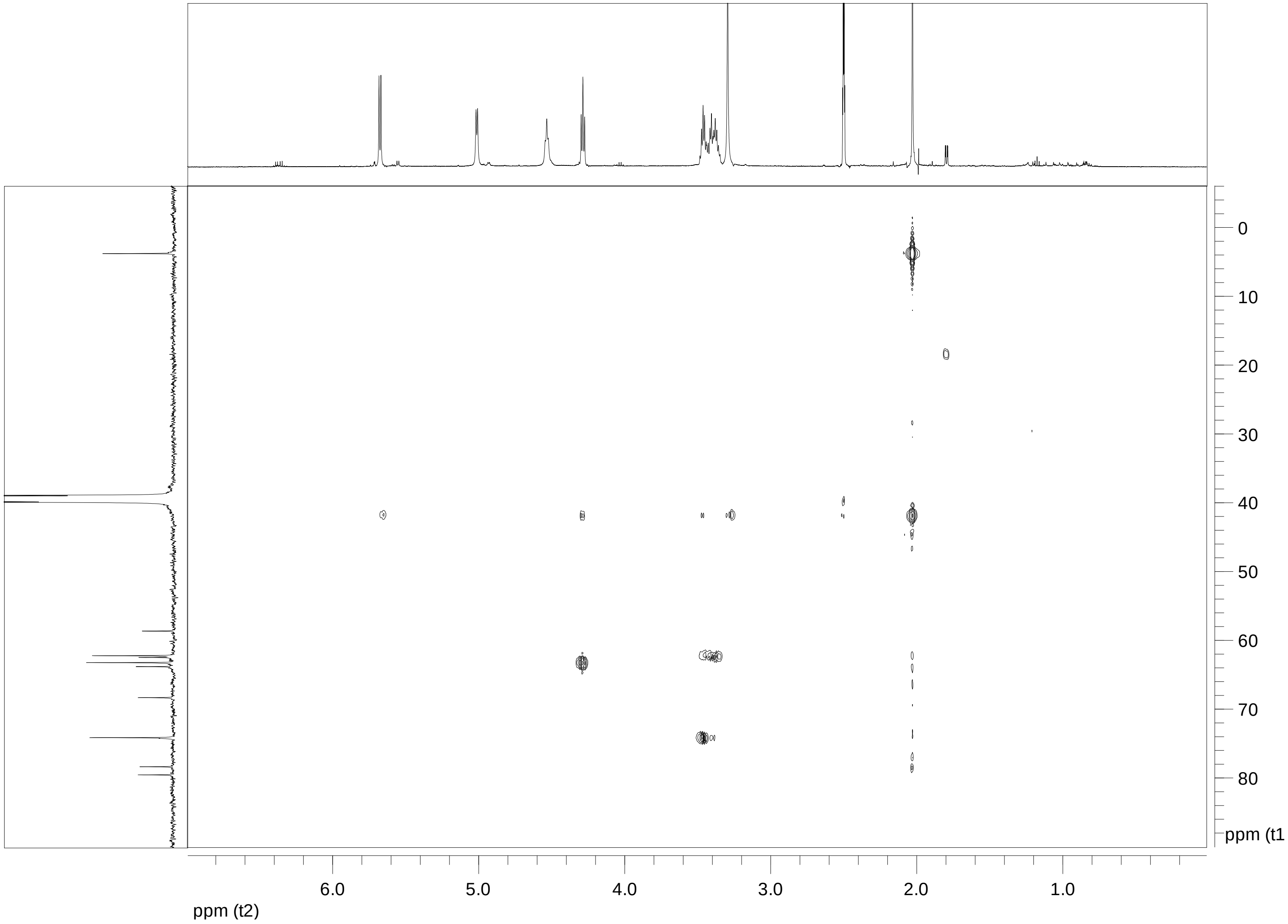

Figure S15. gHMBC spectrum of chondrosterin G (**4**).

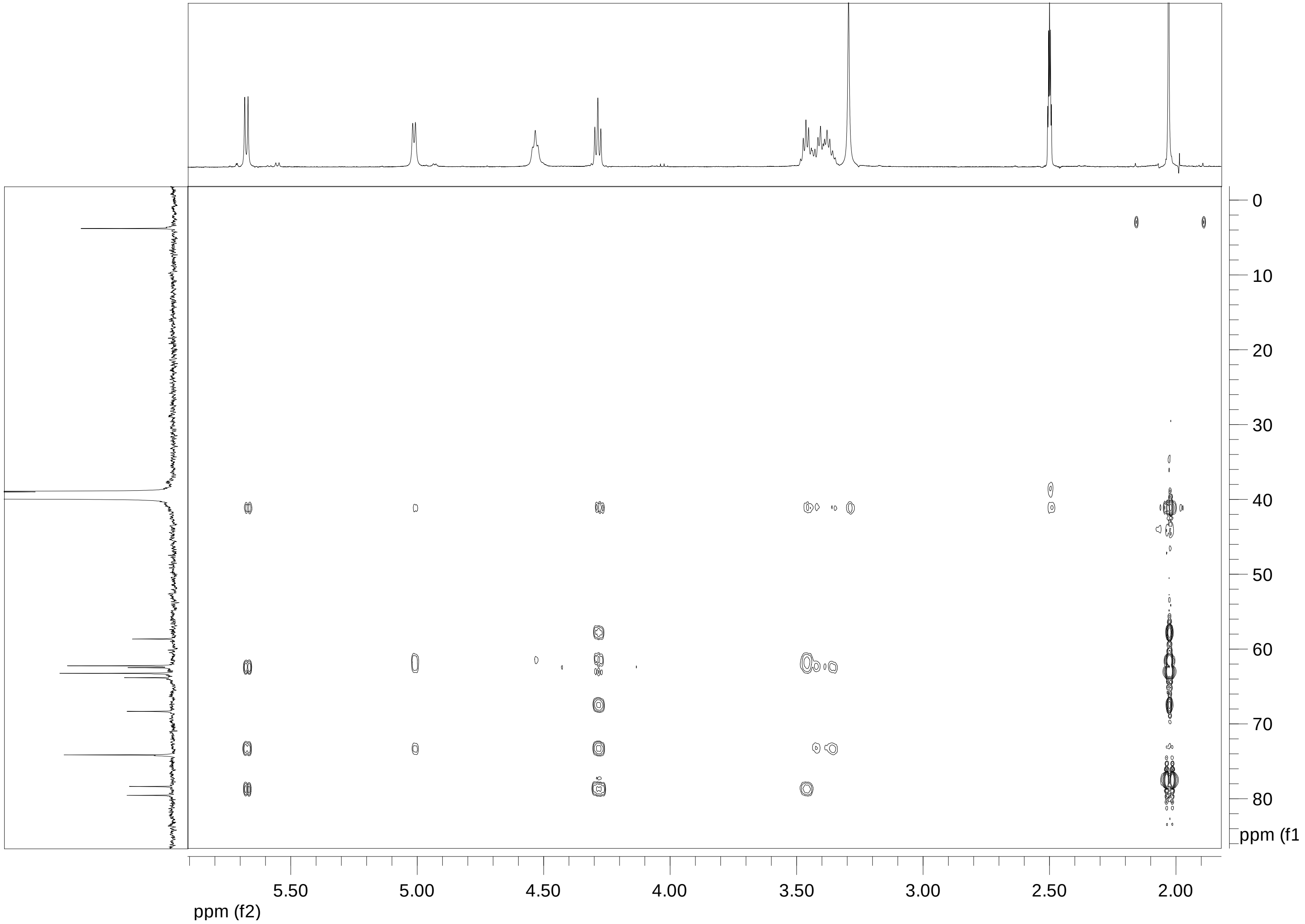

Figure S16. ^{1}H–^{1}H gCOSY spectrum of chondrosterin G (**4**).

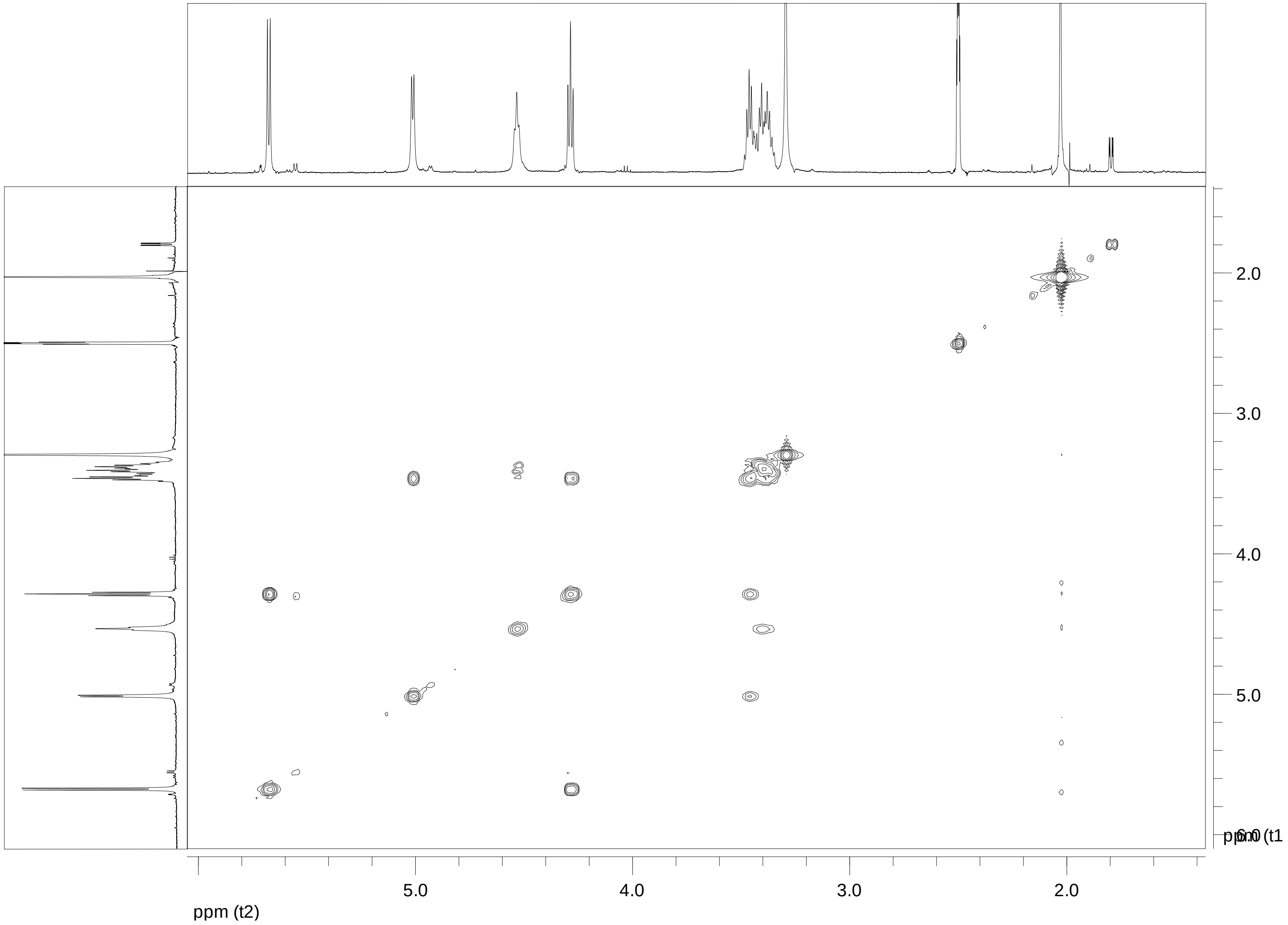

Figure S17. ^{1}H NMR (400 MHz, DMSO-d_6) spectrum of chondrosterin H (**5**).

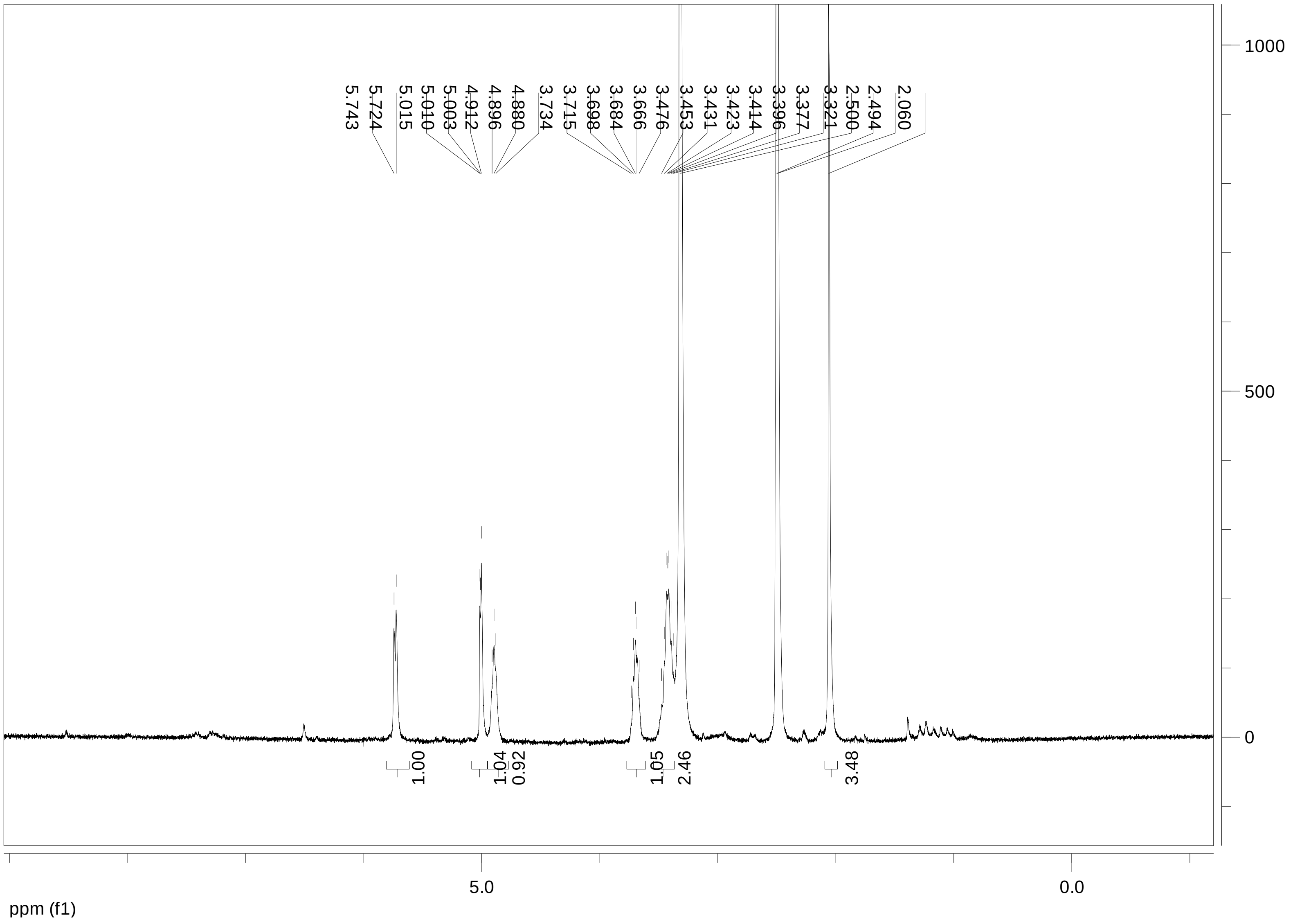

Figure S18. ^{13}C NMR (100 MHz, DMSO-d_6) spectrum of chondrosterin H (**5**).

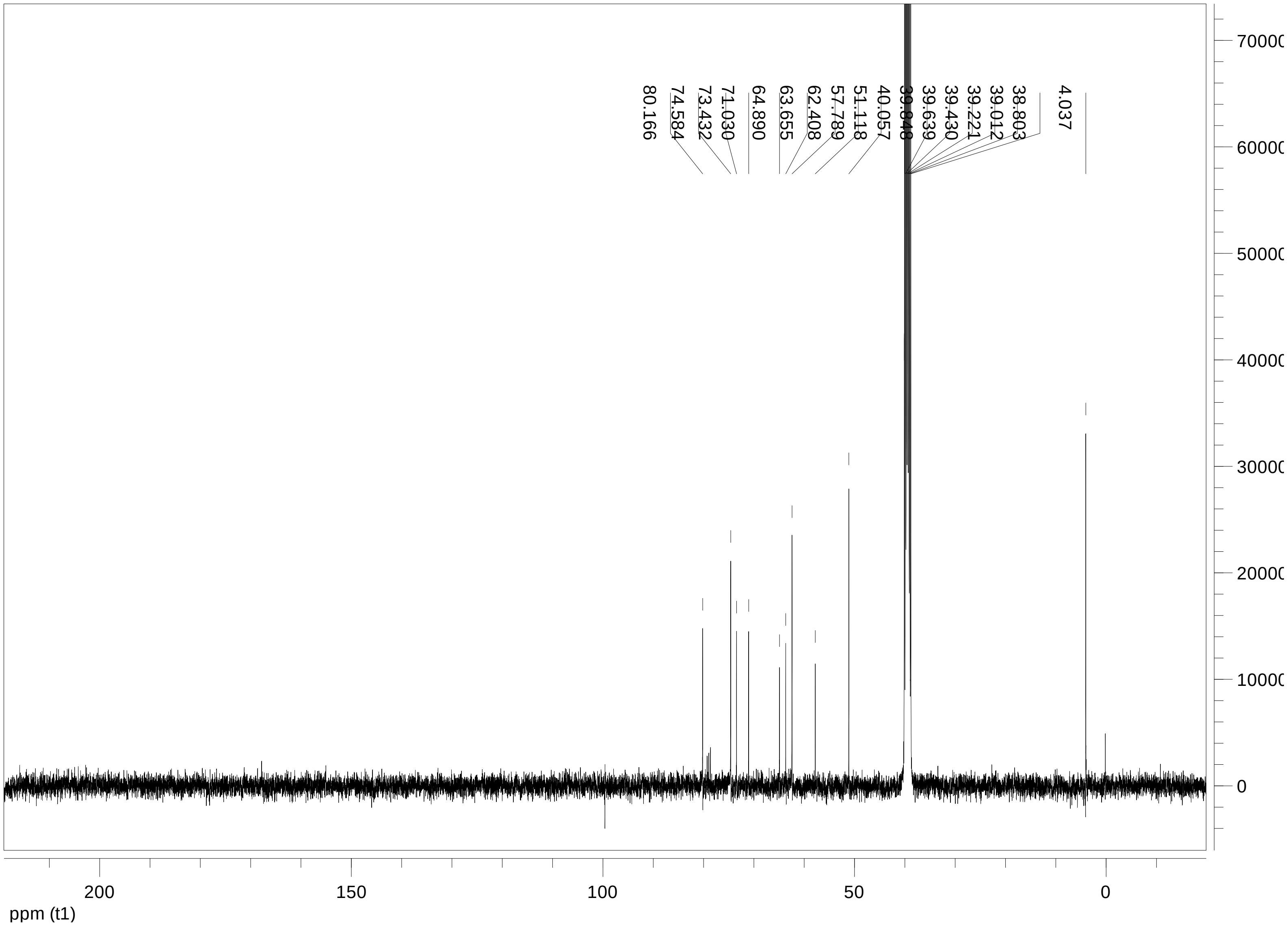

Figure S19. ^{1}H NMR (500 MHz, $CDCl_3$) spectrum of chondrosterin H (**5**).

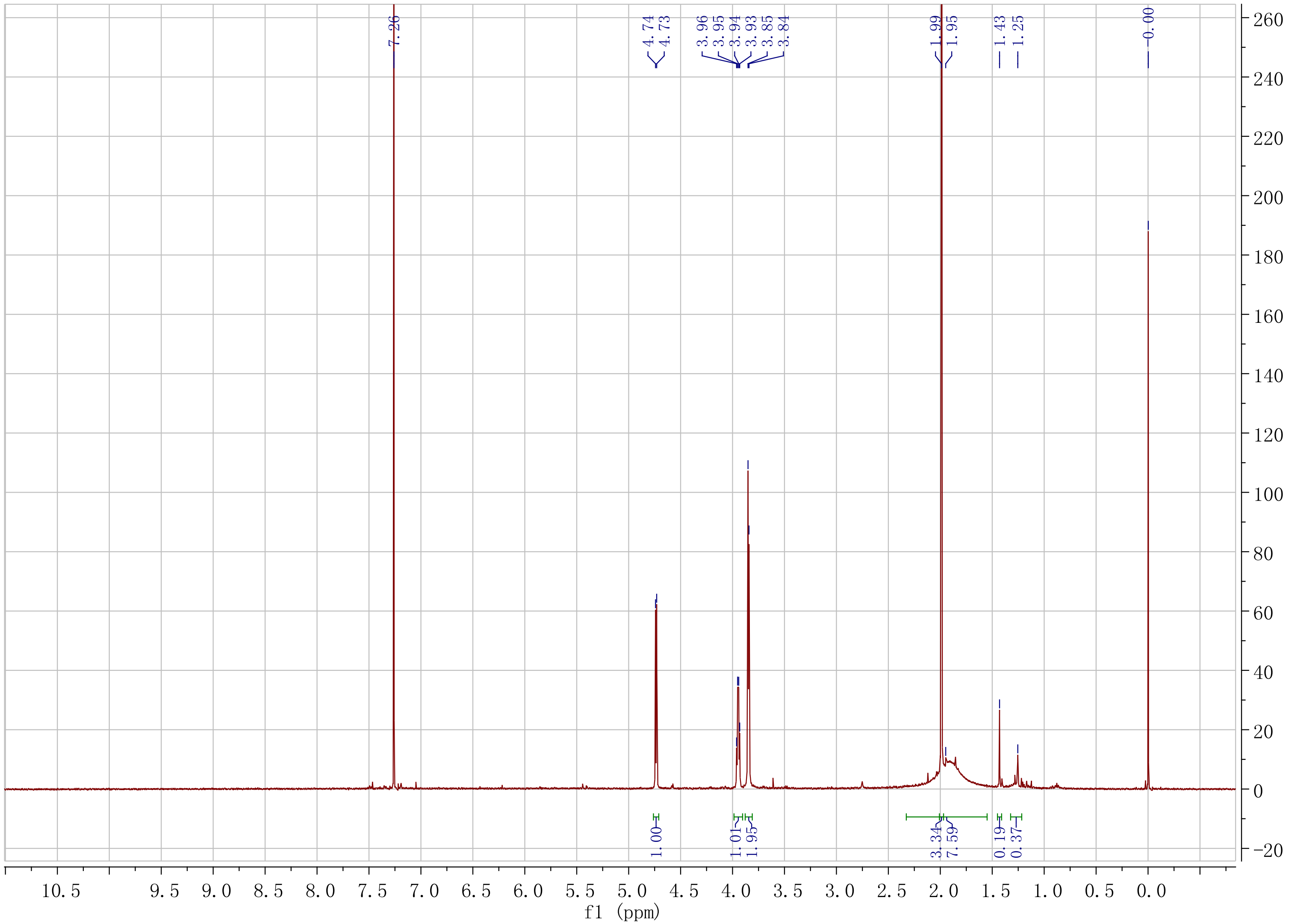

Figure S20. ^{13}C NMR (125 MHz, $CDCl_3$) spectrum of chondrosterin H (**5**).

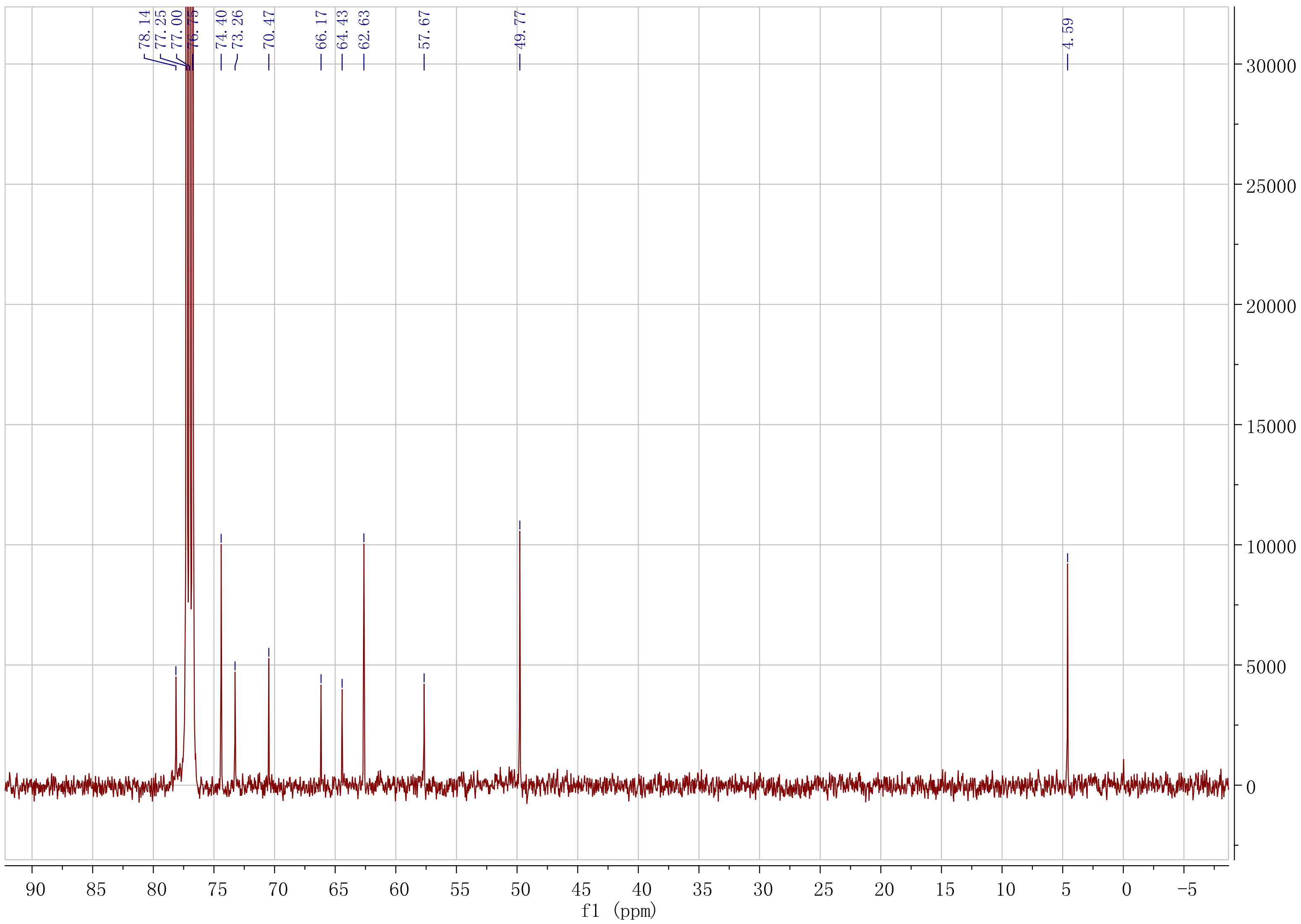

Figure S21. ^{1}H NMR (500 MHz, $CDCl_3$) spectrum of dehydromatricarianol (**6**).

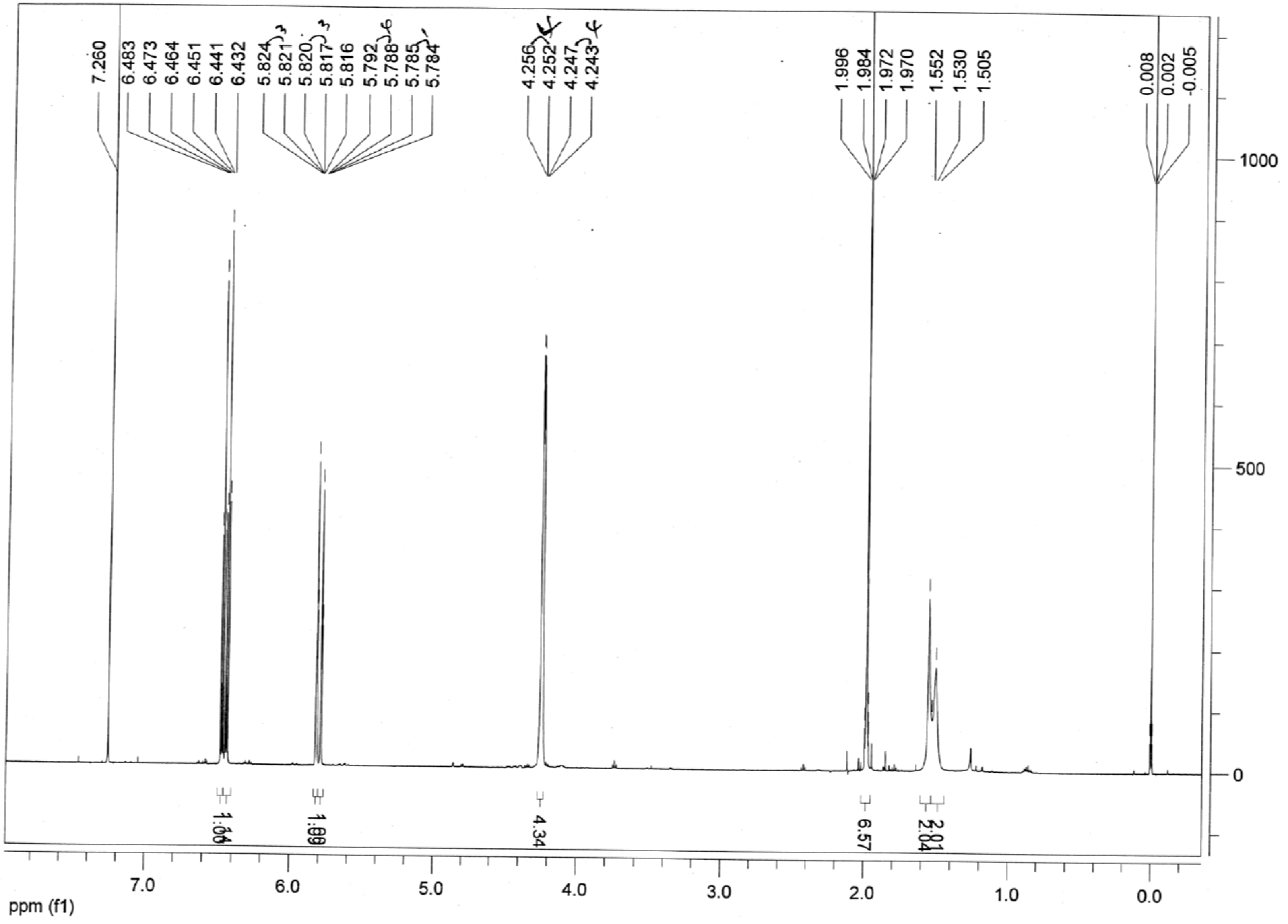

Figure S22. ^{13}C NMR (125 MHz, $CDCl_3$) spectrum of dehydromatricarianol (**6**).

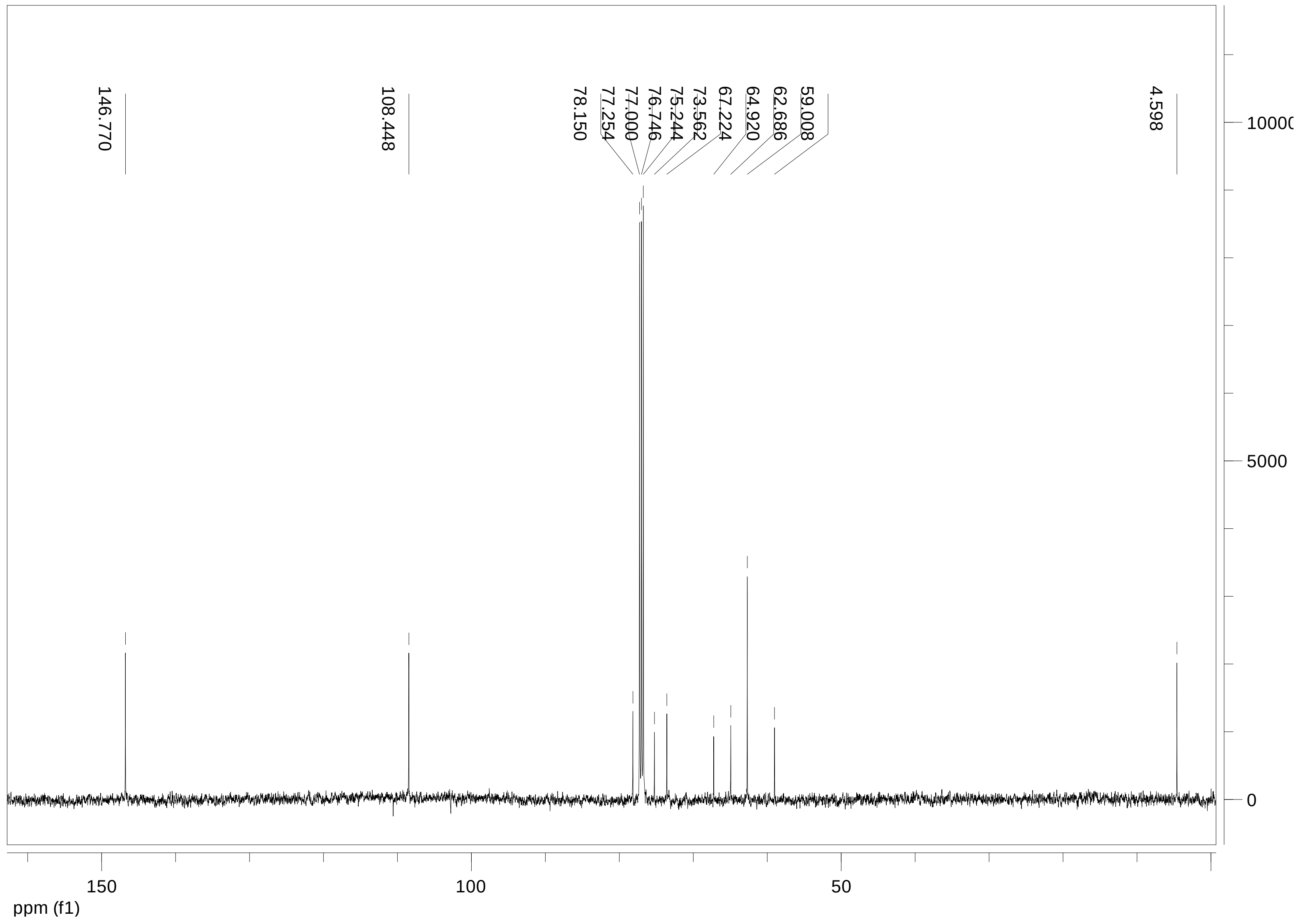

Supplement: Supplementary File 1 — Supplementary Information (PDF, 3702 KB) [file marinedrugs-11-00551-s001.pdf]
